# Impaired branched-chain amino acid (BCAA) catabolism during adipocyte differentiation decreases glycolytic flux

**DOI:** 10.1016/j.jbc.2024.108004

**Published:** 2024-11-16

**Authors:** Courtney R. Green, Lynn M. Alaeddine, Karl A. Wessendorf-Rodriguez, Rory Turner, Merve Elmastas, Justin D. Hover, Anne N. Murphy, Mikael Ryden, Niklas Mejhert, Christian M. Metallo, Martina Wallace

**Affiliations:** 1 Molecular and Cellular Biology Laboratory, Salk Institute for Biological Studies, La Jolla, California, USA; 2 Department of Bioengineering, University of California San Diego, La Jolla, California, USA; 3 Department of Medicine (Huddinge), Karolinska Institutet, ME Endokrinologi, Karolinska University Hospital Huddinge, Huddinge, Sweden; 4 School of Agriculture and Food Science, University College Dublin, Belfield, Ireland; 5 Conway Institute of Biomolecular and Biomedical Research, University College Dublin, Belfield, Ireland; 6 Department of Pharmacology, University of California San Diego, La Jolla, California, USA; 7 Steno Diabetes Center Copenhagen, Herlev, Denmark

**Keywords:** branched chain amino acids, glycolysis, adipogenesis, adipose, metabolic flux

## Abstract

Dysregulated branched-chain amino acid (BCAA) metabolism has emerged as a key metabolic feature associated with the obese insulin-resistant state, and adipose BCAA catabolism is decreased in this context. BCAA catabolism is upregulated early in adipogenesis, but the impact of suppressing this pathway on the broader metabolic functions of the resultant adipocyte remains unclear. Here, we use CRISPR/Cas9 to decrease BCKDHA in 3T3-L1 and human pre-adipocytes, and ACAD8 in 3T3-L1 pre-adipocytes to induce a deficiency in BCAA catabolism through differentiation. We characterize the transcriptional and metabolic phenotype of 3T1-L1 cells using RNAseq and ^13^C metabolic flux analysis within a network spanning glycolysis, tricarboxylic acid (TCA) metabolism, BCAA catabolism, and fatty acid synthesis. While lipid droplet accumulation is maintained in *Bckdha*-deficient adipocytes, they display a more fibroblast-like transcriptional signature. In contrast, *Acad8* deficiency minimally impacts gene expression. Decreased glycolytic flux emerges as the most distinct metabolic feature of 3T3-L1 *Bckdha*-deficient cells, accompanied by a ∼40% decrease in lactate secretion, yet pyruvate oxidation and utilization for *de novo* lipogenesis is increased to compensate for the loss of BCAA carbon. Deletion of BCKDHA in human adipocyte progenitors also led to a decrease in glucose uptake and lactate secretion; however, these cells did not upregulate pyruvate utilization, and lipid droplet accumulation and expression of adipocyte differentiation markers was decreased in BCKDH knockout cells. Overall our data suggest that human adipocyte differentiation may be more sensitive to the impact of decreased BCKDH activity than 3T3-L1 cells and that both metabolic and regulatory cross-talk exist between BCAA catabolism and glycolysis in adipocytes. Suppression of BCAA catabolism associated with metabolic syndrome may result in a metabolically compromised adipocyte.

The branched-chain amino acids (BCAAs) leucine, isoleucine, and valine are essential dietary amino acids whose elevated circulating levels are emerging as a hallmark of metabolic syndrome (1, 2, 3). It has become evident that BCAA metabolism is dysregulated in multiple different tissues in this context and this includes adipose tissue where transcriptional and proteomic studies in humans have shown that BCAA catabolism is significantly suppressed with obesity and insulin resistance (4, 5). In addition, in cross-tissue comparisons of BCAA catabolic flux in lean and obese mice, adipose tissue displays one of the biggest decreases in BCAA breakdown (6, 7). The BCAA pathway is one of the most consistently altered metabolic pathways in the context of adipose dysfunction and thus it is important to develop our knowledge of the role this pathway plays in adipose tissue.

BCAA catabolism is upregulated during adipocyte differentiation (4, 8, 9, 10, 11, 12, 13, 14, 15) and it is initiated by mitochondrial BCAT2, which transfers the amino nitrogen to α-ketoglutarate yielding glutamate and a corresponding branched-chain α-keto acid (BCKA). These BCKAs are subsequently oxidized by the branched-chain ketoacid dehydrogenase (BCKDH) complex, a highly regulated, rate-limiting step in BCAA catabolism. A series of enzymatic steps further catabolize short branched-chain fatty acid (SBCFA)-CoAs to acetyl-CoA and succinyl-CoA which can enter the TCA cycle. In addition to serving as a source of nitrogen and carbon for the cell, many intermediates of BCAA catabolism have been proposed to play signaling roles or serve as post-translational modifications and thus BCAA catabolism may impact cell function in multiple ways (7, 16, 17, 18, 19).

We and others have previously investigated how genetically altering BCAA metabolism impacts adipocyte differentiation and found that shRNA-mediated knockdown of *Bcat2* or *Bckdha* (14), the leucine catabolic enzymes *Mccc1* (11), and siRNA-mediated knockdown of the valine catabolic enzyme *Hibch* (20) throughout differentiation decreased lipid accumulation indicating impaired differentiation. In mice, disruption of BCAA catabolism in white adipocytes *via* knockout has yielded varying results in overall phenotype but enhanced BCAA catabolism has generally been associated with increased white adipose depot size in response to high fat diet (17, 21).

Collectively, these data indicate altered BCAA catabolism impacts adipogenesis and adipose mass but a comprehensive assessment of how this impacts broader metabolism in adipocytes is lacking. Here we use CRISPR/Cas9 to generate pre-adipocytes deficient in *Bckdha* or *Acad8*, followed by metabolic characterization of resultant adipocytes using RNAseq, stable isotope tracing, and metabolic flux analysis. We find that glucose uptake is substantially decreased indicating crosstalk between BCAA catabolism and signaling mechanisms necessary to upregulate glycolysis during differentiation. While *de novo* lipogenesis is maintained in 3T3-L1 adipocytes *via* enhanced pyruvate utilization, human adipocytes do not increase pyruvate utilization and have decreased lipid droplet accumulation and expression of differentiation markers. This study demonstrates how BCAA catabolism during adipogenesis is required for the generation of metabolically competent adipocytes.

## Results

### *Bckdha* deficient 3T3L1 adipocytes maintain lipid droplet formation but display a metabolically compromised transcriptional profile

**Figure 1:**
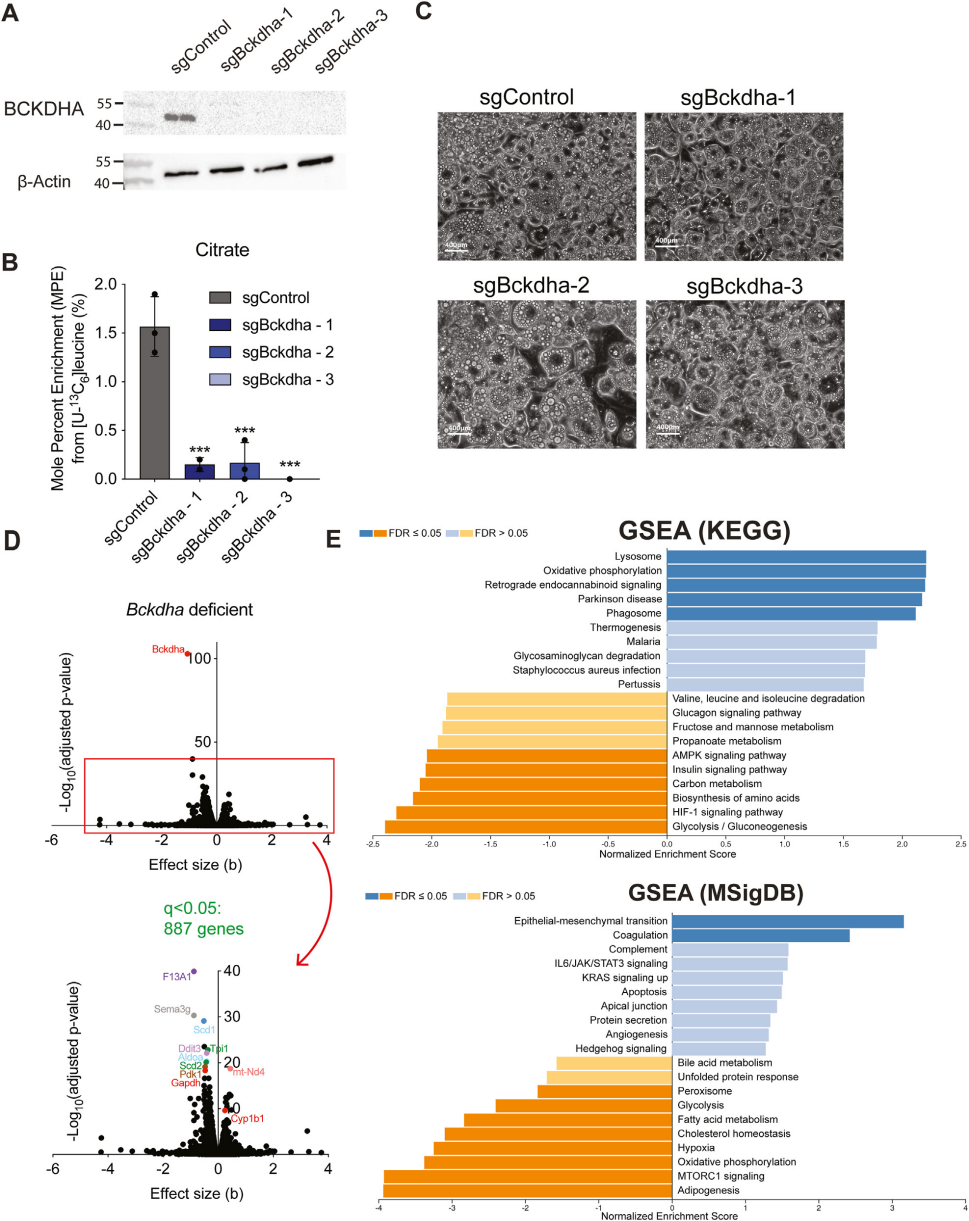
***Bckdha* deficient adipocytes maintain lipid droplet formation but display a metabolically compromised transcriptional profile.** Characterization of sgControl and sgBckdha adipocytes 7 days post-induction of differentiation. *A*, Western blot of BCKDHA levels. *B*, brightfield images (scale bar = 400 μm). *C*, Mole percent enrichment of citrate from [U-^13^C_6_]leucine (means ± SEM, n = 3, analysed *via* one-way ANOVA with Tukeys *post hoc* analysis, ∗∗∗*p* < 0.001 from *post hoc* analysis when compared to sgControl.). *D*, Volcano plots of differentially expressed genes in *Bckdha*-deficient adipocytes compared to Control adipocytes. *E*, gene set enrichment analysis of *Bckdha*-deficient adipocytes generated using WEB-based GEne SeT AnaLysis Toolkit (WebGestalt). *A*–*C*, results are depicted from one representative experiment which was repeated independently at least three times.

To explore the impact of compromised BCAA catabolism in adipocytes, we generated polyclonal cultures of 3T3-L1 pre-adipocytes lacking *Bckdha* using CRISPR/Cas9 and then differentiated these using 3-isobutyl-1-methylxanthine (IBMX), dexamethasone, and insulin. BCKDHA was undetectable in these polyclonal cultures and we observed a 90%+ decrease in incorporation of [U-^13^C_6_]leucine in citrate (Fig. 1, *A* and *B*), along with a decrease in BCAA uptake (Fig. S1*A*). There was no apparent morphological difference with *Bckdha* deficiency, lipid droplet accumulation appeared unaffected (Fig. 1*C*) and mRNA expression of key adipocyte marker genes was largely unchanged in these polyclonal cultures (Fig. S1*B*). To further examine the impact of *Bckdha* deficiency, we performed RNAseq on our polyclonal cell populations. We found 887 genes that were significantly differentially expressed (Fig. 1*D*). The most significantly different genes included *F13a1* and *Sema3g*, which have previously been associated with adipogenesis and obesity (22, 23). To categorize these differentially expressed genes further, we used a WEB-based gene set analysis toolkit (WebGestalt) to perform gene set enrichment analysis (GSEA) with the KEGG pathway functional database. We found seven significantly altered pathways with FDR <0.05, which comprised key functional, metabolic, and signaling pathways relevant to adipocyte function, including oxidative phosphorylation, thermogenesis, glycolysis, HIF-1 signaling, and AMPK signaling (Fig. 1*E*). We also performed GSEA using the MSigDB biological processes hallmark gene sets database. Notably, adipogenesis was the most downregulated pathway in this analysis, and epithelial-to-mesenchymal transition (EMT) was significantly upregulated (Fig. 1*E*), highlighting how *Bckdha* deficiency impacts cellular differentiation state even though lipid droplet accumulation is unchanged.

In addition to *Bckdha* deficiency, which prevents catabolism of all three BCAAs and downstream pathways, we also investigated the phenotype of adipocytes deficient in *Acad8*, which catalyzes the oxidation of valine-derived isobutyryl-CoA (and thus only blocks valine catabolism). ACAD8 protein knockdown (Fig. S1*C*) did not grossly affect lipid accumulation or adipogenic differentiation (Fig. S1*D*). In addition, RNAseq analysis of these *Acad8*-deficient adipocytes found only 65 differentially expressed genes (q < 0.05) (Fig. S1*E*). Overall, these results suggest that *Acad8* deficiency and thus valine valine-specific metabolism elicits more moderate effects compared to *Bckdha* deficiency and what has previously been reported for genes associated with leucine catabolism (11). Therefore, next, we focused on characterizing how metabolism is altered in *Bckdha*-deficient adipocytes.

### *Bckdha*-deficient adipocytes reprogram central carbon metabolism

**Figure 2:**
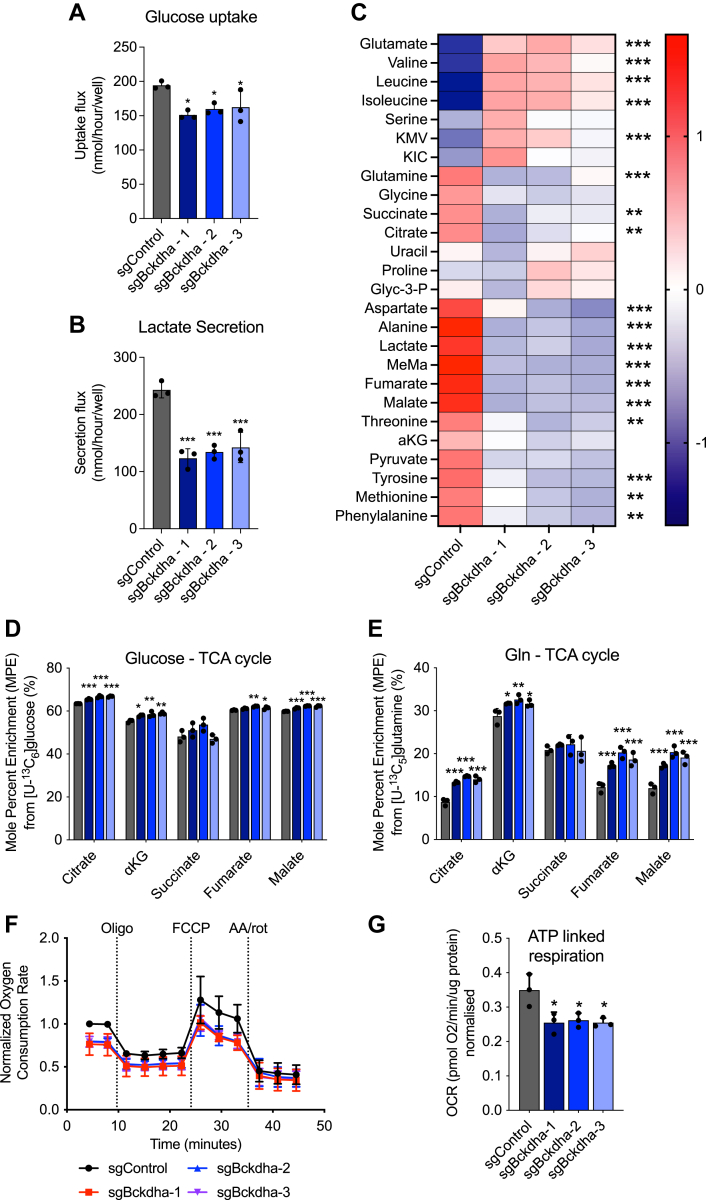
***Bckdha* deficient adipocytes reprogram central carbon metabolism.***A*, molar amount of glucose uptake in Control or *Bckdha*-deficient adipocytes over 48 h. *B*, molar amount of lactate secretion in Control or *Bckdha*-deficient adipocytes over 48 h. *C*, heat map of the abundance of intracellular metabolites in *Bckdha*-deficient adipocytes, statistical analysis *via* one-way ANOVA (9 cellular replicates). FDR∗<0.05, ∗∗<0.01, ∗∗∗<0.001. *D*, mole percent enrichment (MPE) of TCA cycle intermediates after 48 h of incubation with [U-^13^C_6_]glucose. *E* MPE of TCA cycle intermediates after 48 h of incubation with [U-^13^C_5_]glutamine. *F*, normalized oxygen consumption rate of 3T3-L1 Control or *Bckdha*-deficient adipocytes treated with the indicated pharmacological inhibitors (n = 3 experiments internally normalized to sgControl). *G*, ATP-linked respiration (n = 3 individual experiments internally normalized to sgControl). *A*, *B*, *D* and *E*, data are presented as means ± SD with three cellular replicates. Results are depicted from one representative experiment which was repeated independently at least three times. (*A*, *B*, *D*–*G*) One-way ANOVA with Tukeys *post hoc* analysis for comparison of each groups means. Significance in all is from *post hoc* analysis and compared to sgControl. ∗*p* < 0.05, ∗∗*p* < 0.01, ∗∗∗*p* < 0.001.

Glycolysis/gluconeogenesis was one of the most downregulated pathways in our GSEA of *Bckdha*-deficient adipocytes. To explore these changes more directly, we carried out intracellular metabolite analysis and quantified glucose uptake in Control and *Bckdha*-deficient adipocytes. Glucose uptake and lactate secretion were significantly decreased in *Bckdha*-deficient adipocytes (Fig. 2, *A* and *B*) supporting the reduction in glycolysis suggested by our RNAseq analysis. In our intracellular metabolite analysis, pyruvate and TCA cycle intermediates along with the non-essential amino acids (NEAA) alanine, aspartate, and glutamine were significantly decreased in *Bckdha*-deficient adipocytes (Figs. 2*C* and S2*A*). The primary metabolites increased were BCAAs, glutamate, and BCKAs. To determine how impaired BCAA catabolism impacts the use of other carbon sources, we traced adipocytes with [U-^13^C_6_]glucose and [U-^13^C_5_]glutamine and quantified isotope enrichment in TCA intermediates. Importantly, we observed a significant increase in mole percent enrichment (MPE) of citrate, α-ketoglutarate (aKG), and succinate from [U-^13^C_6_]glucose in *Bckdha*-deficient adipocytes (Fig. 2*D*), with a relative increase in the M5 and M6 fractional amount in the isotopologue distribution of citrate which may indicate increased TCA cycle turnover (Fig. S2*B*). MPE from [U-^13^C_5_]glutamine was significantly higher in all TCA intermediates except succinate in *Bckdha*-deficient adipocytes (Fig. 2*E*). Analysis of oxygen consumption rates (OCR) in these cells in response to oligomycin indicated that the *Bckdha*-deficient cells have a significant decrease in ATP-linked respiration (Fig. 2, *F* and *G*); however the basal and maximal OCR were not significantly affected (Fig. S2, *C* and *D*). Collectively this indicates that ablation of BCAA catabolism in adipocytes is marked by compensatory increases in pyruvate and glutamine entry into the TCA cycle which minimizes the impact on mitochondrial respiration.

**Figure 3:**
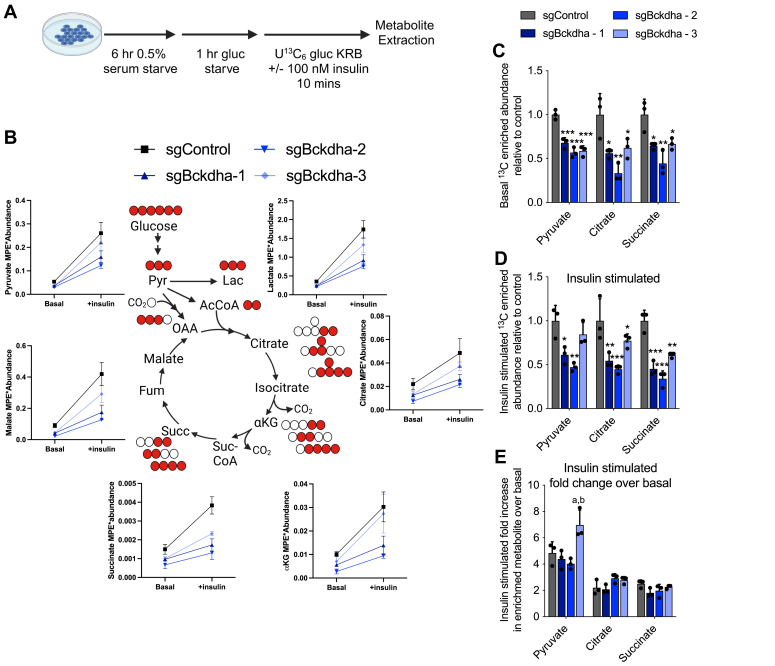
**Insulin-stimulated glucose uptake in *Bckdha* deficient cells.***A*, schematic of experimental design. *B*–*D*, levels of ^13^C enriched metabolite from [U-^13^C_6_]glucose in cells under basal and insulin-stimulated conditions. *E*, fold increase in ^13^C enriched metabolite between basal and insulin-stimulated conditions. Data are presented as means ± SD with three cellular replicates. One-way ANOVA with Tukey *post hoc* analysis for comparison of each group means. ∗*p* < 0.05, ∗∗*p* < 0.01, ∗∗∗*p* < 0.001 indicates comparison to sgControl. ^a^p < 0.05 indicates comparison to sgBckdha-1 and ^b^p < 0.05 indicates comparison to sgBckdha-2.

Our metabolic studies were carried out under nutrient-replete conditions; however, glucose utilization in adipocytes is often functionally assessed *via* acute insulin-stimulated conditions in media depleted of nutrients other than glucose. To understand how glucose metabolism is changed in these conditions, we traced cells for 10 min in [U-^13^C_6_]glucose ± insulin following incubation in media depleted of serum and glucose (Fig. 3*A*). We found a general decrease in total ^13^C-labelled pyruvate and TCA intermediates under both basal(-insulin) and insulin-stimulated conditions indicating a general decrease in glucose utilization is retained and potentially exasperated under these conditions (Fig. 3, *B*–*D*). However, the fold change between basal and insulin-stimulated was not decreased in *Bckdha*-deficient adipocytes (Fig. 3*E*) indicating altered glucose metabolism is unlikely to arise from a change in insulin signaling in these cells but rather a general decrease in glycolytic capacity.

### *Bckdha* deficiency alters lipogenic acetyl-CoA sourcing and fatty acid (FA) diversity

**Figure 4:**
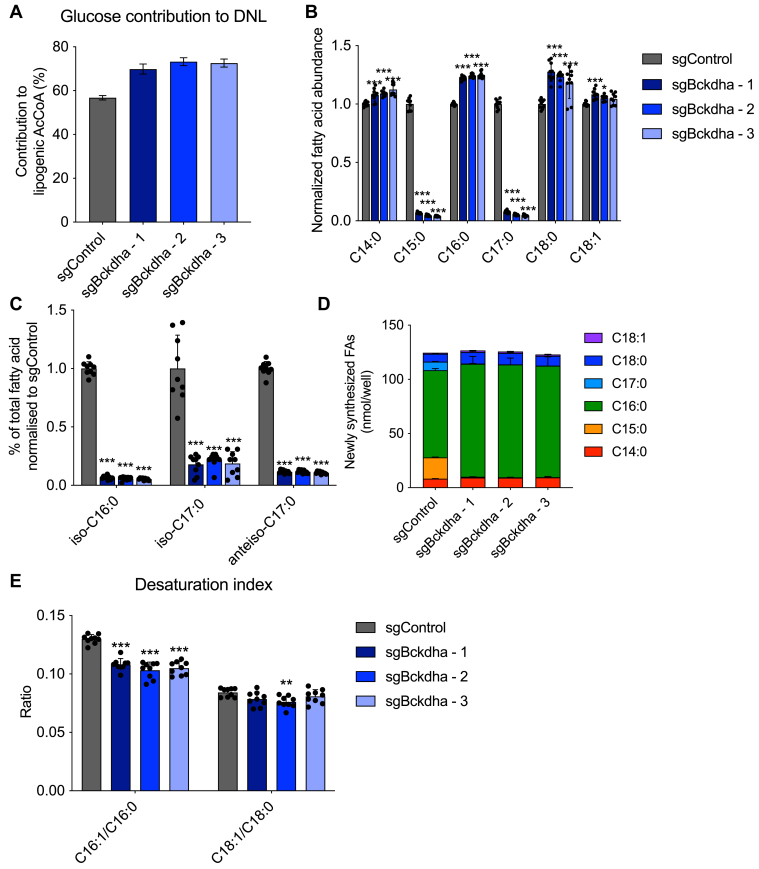
**Bckdha deficiency alters lipogenic acetyl-CoA sourcing and fatty acid diversity.***A*, contribution of [U-^13^C_6_]glucose to the lipogenic AcCoA pool used for *de novo* lipogenesis (DNL) as determined *via* ISA. *B*, relative fold change in total odd- and even-straight-chain fatty acids in *Bckdha*-deficient 3T3-L1 adipocytes normalized to Control adipocytes. *C*, relative fold change in total branched-chain fatty acids (BCFAs) in *Bckdha*-deficient 3T3-L1 adipocytes normalized to Control adipocytes. *D*, molar amount of indicated fatty acids synthesized over 48 h obtained by combining individual FA DNL values with pool size. *E*, desaturation index in Control and *Bckdha*-deficient 3T3-L1 adipocytes. Data are presented as means ± SD (*B*–*D*), means with 95% confidence interval (C.I.) (*A*), and means ± SEM (*E*) with three cellular replicates. Each parameter was analysed *via* one-way ANOVA with Tukeys *post hoc* analysis except A, where significance is denoted as non-overlapping 95% C.I. Significance in all is compared to sgControl. Results are depicted from one representative experiment which was repeated independently at least three times. ∗*p* < 0.05, ∗∗*p* < 0.01, ∗∗∗*p* < 0.001.

Given that lipid droplet accumulation was relatively unaffected by *Bckdha* deficiency and that our previous work demonstrated that BCAAs are a significant source of lipogenic acetyl-CoA in adipocytes (14), we sought to determine whether glucose utilization for *de novo* fatty acid synthesis was altered. Isotopomer spectral analysis (ISA) of palmitate labeling from [U-^13^C_6_]glucose demonstrated that the contribution of glucose derived carbon to the lipogenic acetyl-CoA pool was significantly increased despite the observed decrease in glucose uptake (Fig. 4*A*). 3T3-L1 adipocytes typically use BCAA-derived propionyl-CoA and branched-CoAs to synthesize odd-chain fatty acids (OCFAs) and monomethyl branched-chain fatty acids (mmBCFAs) *via* FASN in addition to palmitate (7, 9, 14). As expected, OCFAs and mmBCFAs were greatly decreased (Fig. 4, *B* and *C*), while the proportion of even-chain fatty acids was significantly increased in *Bckdha*-deficient adipocytes (Fig. S3*C*). ISA modeling also revealed a decrease in newly synthesized OCFAs and a modest increase in the proportion of newly synthesized C16:0 (Figs. 4*D* and S3*A*). However, the total amount of newly synthesized fatty acids (accounting for both the percentage of pool newly synthesized and molar amount of FA) remained unchanged in *Bckdha*-deficient adipocytes (Fig. 4*D*). These results indicate that central carbon metabolism is rewired to sustain FA synthesis (*i.e.*, acetyl-CoA sourcing) and overall abundances in 3T3-L1 adipocytes.

Our RNAseq results also highlighted a decrease in the expression of the desaturase enzymes SCD1 and SCD2 in *Bckdha*-deficient adipocytes. To this end, we calculated the desaturation index (ratio of monounsaturated to saturated FA) in each cell type. While the C18:1/C18:0 ratio was unchanged, the C16:1/C16:0 ratio was significantly decreased in *Bckdha*-deficient adipocytes indicating a decrease in C16:0 desaturation. As both C18:1 and C18:0 can be derived more from uptake than synthesis (compared to C16:1 and C16:0), this difference likely reflects a decrease in desaturation activity that is buffered by import of C18:1 from serum in media (Fig. 4*E*).

### MFA modeling of *Bckdha*-deficient adipocytes

**Figure 5:**
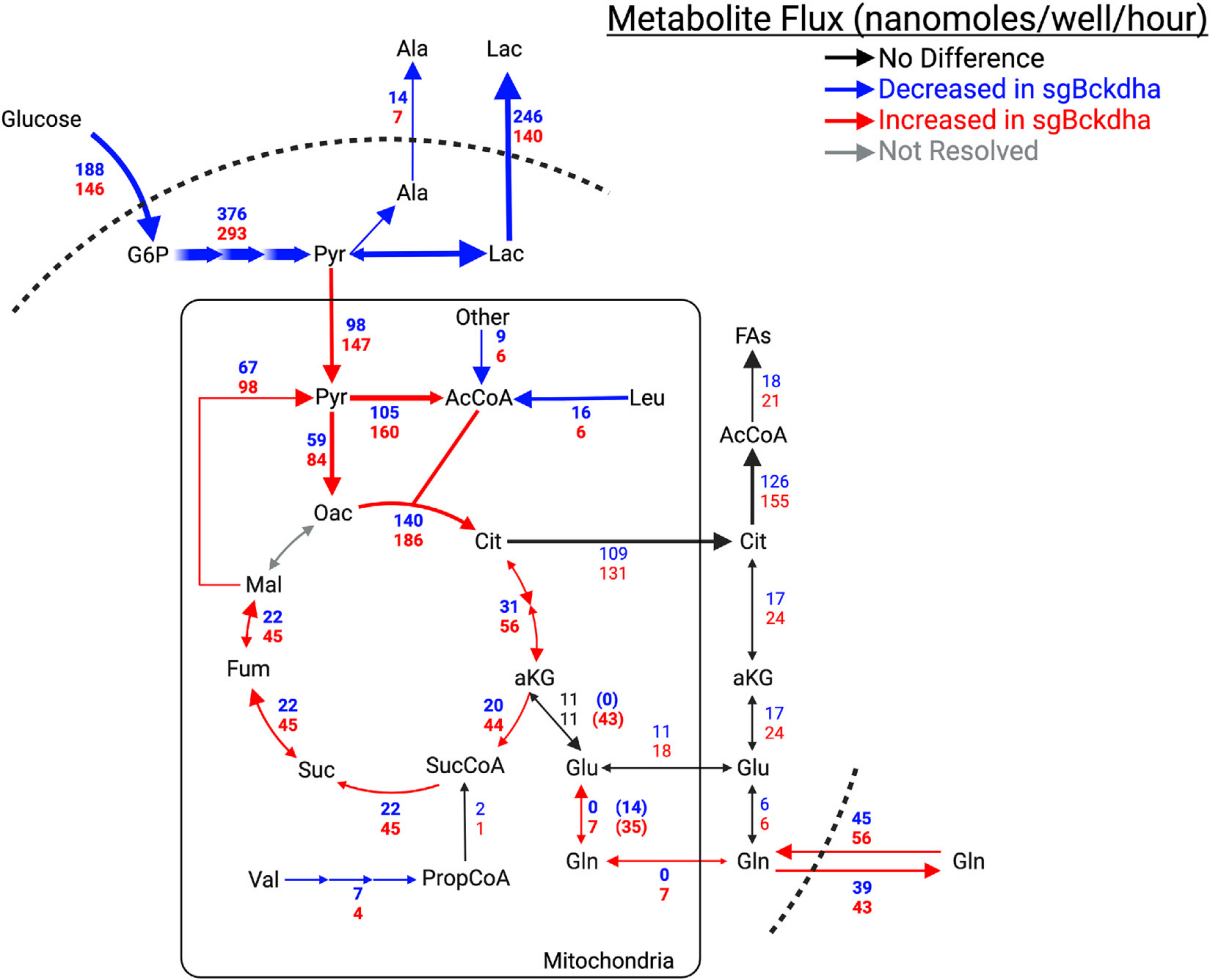
**Metabolic flux analysis reveals significant reprogramming of glycolysis and TCA metabolism.** Map of core MFA model. Values are presented as net flux (exchange flux). The *blue* text is sgControl, *red* text is sgBckdha. *Blue arrows* indicate a flux is decreased in sgBckdha compared to sgControl, while a *red arrow* indicates an increase in flux in sgBckdha. *Bold* text indicates significantly different reactions.

As glucose uptake was decreased, yet utilization of glucose for *de novo*-made fatty acids was increased, we developed a ^13^C metabolic flux analysis (^13^C-MFA) model incorporating central carbon metabolism and lipid biosynthesis to better understand how glucose metabolism was being altered. We incorporated data from Control and *Bckdha*-deficient adipocytes cultured with either [U-^13^C_6_]glucose, [U-^13^C_6_]leucine, or [U-^13^C_5_]valine for 48 h into a MFA model using INCA (24). The network encompassed relevant reactions within glycolysis, TCA metabolism, BCAA oxidation, and fatty acid synthesis, with compartmentalization of several pathways included (Fig. 5). Isotope enrichment data for pyruvate, TCA cycle intermediates, intracellular and extracellular glutamine, intracellular glutamate, leucine, KIC, valine, and fatty acids C15:0, C16:0, and C17:0, as appropriate were included in the model (Table S1) along with direct measurements of uptake and secretion fluxes for glucose, lactate, alanine, valine, leucine, glutamate, and glutamine (Tables S2 and S3).

Flux estimates and confidence from the model highlighted a significant decrease in glucose uptake and both lactate and alanine secretion in 3T3-L1 *Bckdha*-deficient adipocytes. On the other hand, *Bckdha*-deficient adipocytes exhibited a marked increase in pyruvate dehydrogenase (PDH) and pyruvate carboxylase (PC) flux which is consistent with the observed increase in glucose enrichment in TCA intermediates and glucose-derived carbon being used for fatty acid synthesis when acetyl-CoA derived from leucine/isoleucine catabolism is decreased. Indeed, the PDH kinase PDK1 was one of the most significantly downregulated genes in our RNAseq analysis (Fig. 1*D*), indicating there may be decreased inhibitory regulation of PDH in *Bckdha*-deficient adipocytes. We also observed increased pyruvate cycling and although we could not resolve cytosolic from mitochondrial malic enzyme flux, these results highlight the importance of pyruvate cycling in these cells, as the cytosolic malic enzyme is a major producer of lipogenic NADPH in adipocytes (25, 26).

Notably, anaplerotic flux of BCAAs to succinyl-CoA was low in all cells, presumably due to the lack of vitamin B12 present in these cultures (14). This shift in carbon balancing from BCAA oxidation to glucose/pyruvate oxidation resulted in the model predicting an increase in oxidative TCA cycle flux. Indeed, glutamine contribution to the TCA cycle was also estimated to be increased in *Bckdha*-deficient adipocytes, which also exhibited dramatically higher rates of exchange between glutamate and *α*-KG. These results are in agreement with our independent results using [U-^13^C_5_]glutamine as a tracer (Fig. 2*E*), which were not incorporated into the model due to the high exchange with medium glutamine. Notably, the model predicted both an increase in the uptake (56 nmol/well/h vs 45 nmol/well/h) and secretion (43 nmol/well/h vs 39 nmol/well/h) of glutamine resulting in a net decrease in total glutamine in the spent medium. Overall, these findings highlight the broad impact of *Bckdha* deficiency on both central carbon and nitrogen metabolism (27). In this manner, altered BCAA metabolism can have broad impacts on adipose tissue function associated with carbohydrate disposal and amino acid homeostasis.

### *BCKDHA* deficiency in a human adipocyte model

**Figure 6:**
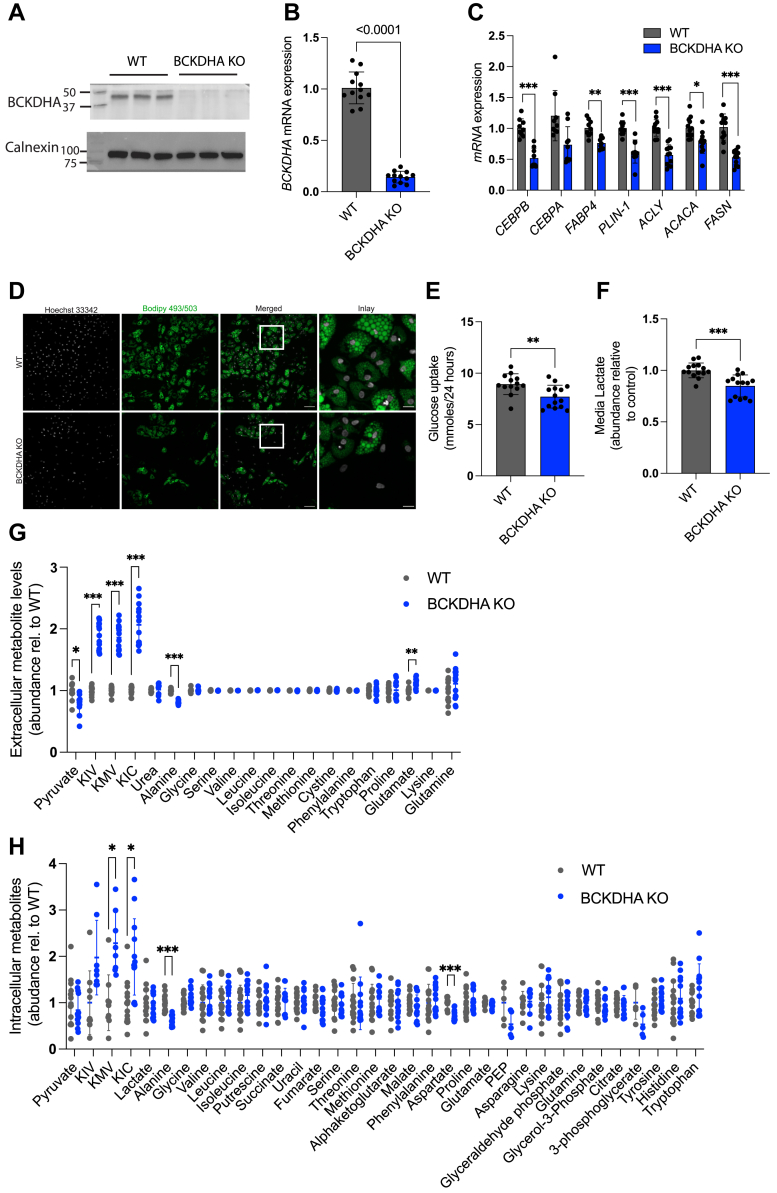
**BCKDH deficiency impacts differentiation and glycolytic flux in human adipocytes.** Characterization of control (WT) and BCKDHA knock-out (KO) human adipocytes. *A*, Western blot of BCKDHA (three cellular replicates). *B*, gene expression of BCKDHA (12 cellular replicates) *C*, gene expression of differentiation markers (9-12 cellular replicates). *D*, bodipy staining of lipid droplets. Scale bars, 100 μm and 25 μm (inlay). *E*, Glucose uptake (13 cellular replicates). F and G Extracellular lactate (14 cellular replicates) and metabolite levels (13 cellular replicates) in media following 24 h of culture. *H*, intracellular metabolite levels 24 h post media change (14 cellular replicates). Data are presented as means ± SD. For A and D, results are depicted from one representative experiment which was repeated independently at least two-three times. For (*B*–*C*) and (*E*–*H*), all cellular replicates were collected over two-three separate differentiation rounds. Two-tailed students *t* test was used to test significant differences in (*B*–*C*) and (*E*–*H*). Multiple unpaired t-tests with correction for multiple comparison using the Holm-Sidak method was used in (*G*–*H*). ∗*p* < 0.05, ∗∗*p* < 0.01, ∗∗∗*p* < 0.001.

To understand how altered BCKDH activity impacts human adipocyte formation, we used Cas9-mediated engineering to generate polyclonal cultures of hAPCs lacking *BCKDHA* (Fig. 6, *A* and *B*), and then differentiated them in serum-free media as previously described (28). Control cells electroporated with a scrambled sgRNA were cultured in parallel (WT). In contrast to the findings in 3T3-L1 cells, we found that gene expression of differentiation markers and lipid droplet accumulation was significantly decreased (Fig. 6, *C* and *D*) in the KO cells. Analysis of metabolite levels revealed a significant decrease in glucose uptake, along with reduced levels of lactate, alanine, and pyruvate in the conditioned media (Fig. 6, *E*–*G*) similar to findings in 3T3-L1 adipocytes (Figs. 2*B* and 5). BCAA uptake did not significantly differ but levels of BCKAs, the direct substrates of BCKDH increased in the media (Fig. 6*G*). An increase in intracellular BCKAs was also found, along with a decrease in alanine and aspartate levels (Fig. 6*H*) in the KO cells. To determine if human adipocytes also upregulated glucose-derived carbon entry into the TCA cycle, we incubated WT and KO cells with [U-^13^C_6_]glucose for 24 h. We found minimal change in total ^13^C incorporation into TCA intermediates between WT and KO cells (Fig. S4*A*). However, the mass isotopomer distribution of ^13^C derived from glucose differed with a decrease in M3 and M5 citrate, and a relative increase in M4 indicating a potential alteration in the relative activity of PDH and pyruvate carboxylase (PC) (Fig. S4*B*). Collectively, these data indicate that BCKDHA deficiency during human adipocyte differentiation has a more pronounced impact on differentiation than in the 3T3-L1 model. While both 3T3-L1 cells and human adipocytes deficient in BCKDHA have decreased glycolytic flux, human adipocytes are not able to compensate *via* increased glucose-derived carbon entry into the TCA cycle.

### *BCKDHA* correlates with the expression of glycolytic genes in human adipose tissue

Finally, to determine whether there is a relationship between the expression of *BCKDHA* and glycolytic gene expression in adipose tissue *in vivo*, we utilized the web app Correlation AnalyzeR which utilizes the ARCHS4 data repository of publicly available RNA-sequencing data (29). Specifically, we took the list of genes driving the “Glycolysis/Gluconeogenesis” KEGG geneset in our RNAseq analysis (Fig. 1*E*) and performed gene vs gene list analysis in adipose tissue. We found that 11 of the 12 input genes were positively correlated (Table S7) and overall, the input gene list was significantly correlated with *BCKDHA*, as determined by comparison against a list of random genes that allows approximation of the likelihood that the primary gene and input gene list are correlated greater than would be predicted by random chance (Fig. S4, *C*–*D*).

## Discussion

Here we explored how BCKDH knockout, which leads to an absence of leucine, isoleucine, and valine catabolism impacts differentiation and metabolism in cultured 3T3-L1 and human adipocytes. We found that adipocyte lipid droplet accumulation and *de novo* lipogenesis can be maintained in the absence of BCKDH in 3T3-L1 cells but this was not the case in human adipocytes where BCKDH KO resulted in cells with decreased lipid droplet accumulation. However, both cell types displayed decreased glucose uptake along with a decrease in alanine and lactate efflux indicating a decrease in glycolytic flux. Overall, our findings indicate that decreased BCAA catabolism during adipogenesis can have a variable impact on markers of differentiation but consistently impacts the metabolic capacity of adipocytes which may prime the cells for further dysfunction in the obese state and impact intercellular metabolite cross-talk in adipose tissue.

In our 3T3-L1 system, we found there was a decrease in glycolysis and lactate secretion under both typical nutrient-replete conditions when cells were monitored over 24 h and acute insulin stimulation. However, the fold difference in glucose utilization between basal and insulin-stimulated conditions was not decreased indicating insulin signaling is not compromised but rather there is a general decrease in glycolytic capacity. This metabolic change was also reflected in the gene expression signature where glycolysis was the most downregulated pathway in our GSEA using the KEGG functional database. This suggests there is cross-talk between BCAA catabolism and signaling mechanisms necessary to regulate glycolysis during adipocyte differentiation. GSEA also indicated a change in both mTORC1 and HIF-1 signaling which are known to regulate glycolysis (30). HIF-1*α* mRNA is increased during adipocyte differentiation (31) and previous studies have found that HIF-1*α* can drive increased glycolysis in white adipocytes when *α*KG levels are decreased due to GLS inhibition (32), and can support glycolytic flux in brown adipocytes (33). The potential functional impact of this in an adipose tissue context is unclear; however, recent studies have suggested lactate production is prioritized by adipocytes (34). In addition, lactate is emerging as an important substrate for fueling TCA cycle metabolism (35) and inter-cellular crosstalk (36). Overall, further studies will be required to understand the cross-talk between BCAA catabolism and glycolysis during adipocyte differentiation and whether suppression of BCAA catabolism during adipogenesis *in vivo* would impact adipose lactate metabolism.

Surprisingly, although glucose uptake was decreased, our data in 3T3L1 adipocytes indicates a significant increase in pyruvate utilization *via* increased flux through both pyruvate dehydrogenase and pyruvate carboxylase. However, this did not occur in human adipocytes. Previous studies have found that elevated BCKAs due to decreased BCKDH activity or *via* exogenous addition of BCKAs inhibit pyruvate dehydrogenase in hepatocytes, the heart, and the brain (37, 38, 39, 40) as well as the mitochondrial pyruvate carrier in hepatocytes (41). Notably, we found that BCKAs were significantly elevated both intracellularly and extracellularly in our human adipocyte BCKDH KO cells and BCAA levels were unaffected. However, BCKA levels were less impacted in our 3T3-L1 cells and instead, these cells decreased BCAA uptake. Therefore altered BCKA accumulation may contribute to the differential impact that BCKDHA KO has in both cell systems on pyruvate metabolism.

BCKDHA deficient 3T3-L1 adipocytes also displayed a significant difference in their fatty acid composition, most notably decreased mmBCFAs and OCFAs which we have previously shown are synthesized from intermediates in the BCAA catabolic pathway (7). mmBCFAs have been proposed to signal *via* mTORC1 (42) and have been implicated in altered immune cell signaling (43, 44) thus altered levels of these metabolites may have a significant impact on intercellular communication with other adipose resident cells *in vivo.*

Finally, we found that decreased BCAA catabolism had a variable impact on markers of adipocyte differentiation dependent on cell type. Lipid droplet accumulation was not impacted in our polyclonal *Bckdha*-deficient 3T3-L1 adipocytes and this contrasts our prior results targeting *Bckdha* using shRNA (14) and those of others utilizing shRNA or siRNA to alter MCCC1 and HIBCH levels (11, 20). However, in our RNAseq analysis using the molecular signature database, adipogenesis was one of the most downregulated pathways along with an increase in the expression of genes associated with epithelial-to-mesenchymal transition indicating a more fibroblast-like gene signature. In BCKDHA-deficient human adipocytes, there was a more pronounced impact on markers of differentiation and we observed decreased lipid droplet accumulation along with decreased gene expression of key markers of differentiation. These differences may be due to a number of factors such as species variation and/or cell culture differences as in contrast to 3T3-L1 cells, the human adipocytes utilized here are cultured in serum-free conditions *versus* 10% FBS in the 3T3-L1 system. Notably, there are many human inborn errors of metabolism in the BCAA catabolic pathway, and lipodystrophy is not noted as a primary feature of those patients with maple syrup urine disease which impacts BCKDH function. However, lipodystrophy has been reported in methylmalonic acidemia patients who have a defect in methylmalonyl CoA mutase which converts intermediates of isoleucine and valine catabolism to succinyl-CoA (45). In mouse studies, the ability to fully probe the impact of decreased BCKDH activity on adipocyte differentiation is challenging as most adipose-specific promoters are not activated at the start of differentiation or are unspecific (46) while whole-body knockouts have been reported as lethal post-weaning (47). Despite these challenges, there are multiple examples *in vivo* where disrupted BCAA catabolism in adipose is associated with systemic dysregulation of metabolism (48, 49). As our knowledge of, and thus ability to genetically target key progenitor populations in adipose tissue increases, it is likely that the role of BCKDH in adipocyte differentiation and metabolism *in vivo* will be further uncovered.

Overall, our studies highlight how BCKDHA deficiency during differentiation re-wires the metabolic network and the levels of multiple metabolites that have been shown to play a role in intracellular cross-talk *in vivo*. However, further studies are needed at multiple time points to understand how altered BCAA catabolism during adipocyte differentiation impacts the signaling networks that regulate transcription of other metabolic pathways and the key nodes and time points where it is most influential.

## Materials and methods

### 3T3-L1 Cell culture and differentiation

All reagents were purchased from Sigma-Aldrich unless otherwise noted. All media and sera were purchased from Life Technologies unless otherwise stated. Mouse 3T3-L1 pre-adipocytes were provided by Prof. Alan Saltiel and cultured in high glucose Dulbecco’s modified Eagle medium (DMEM) (Life Technologies) supplemented with 10% bovine calf serum (BCS) below 70% confluence. Cells were regularly screened for *mycoplasma* contamination. For differentiation, 10,000 cells/well were seeded onto 12-well plates and allowed to reach confluence (termed Day −1). On Day 0, differentiation was induced with 0.5 mM 3-isobutyl-1-methylxanthine (IBMX), 0.25 μM dexamethasone, and 1 μg/ml insulin in DMEM containing 10% FBS. The medium was changed on Day 3 to DMEM + 10% FBS with 1 μg/ml insulin. Day 6, and thereafter, DMEM + 10% FBS was used.

Isotope tracing was carried out 7 days post-induction of differentiation. Cells were incubated in custom DMEM (Hyclone) in which the metabolite specified was replaced with the ^13^C- or ^2^H-labeled version for 24 h unless otherwise specified. Fatty acids were then either calculated as percent total fatty acids and normalized to control conditions or normalized to [^2^H_31_]Palmitate internal standard and normalized to control conditions.

### Generation of lentiviral CRISPR/Cas9 KO 3T3-L1 adipocytes

Control, *Bckdha*, and *Acad8* target sequences (Table S4) were cloned into the lentiCRISPRv2 plasmid, a gift from Feng Zhang (Addgene plasmid #52961) (50). For lentivirus production, 2 to 2.5 million HEK293FT cells were placed in 10 cm tissue culture plates in 2 ml of DMEM (containing 1% penicillin/streptomycin, 10% FBS). 24h later, transfection was performed using Lipofectamine 3000 (Invitrogen) with 1.3 μg VSV.G/PMD2.G, 5.3 μg of lenti-gag/pol/PCMVR8.2 and 4 μg of lentiviral vector. Lentivirus-containing supernatants were harvested 48 and 72h later, combined, and concentrated using Amicon Ultra-15 centrifugal filters, 100,000 NMWL (Millipore) following the manufacturer’s protocol. 3T3-L1 pre-adipocytes were infected with 7 μl of virus in 2 ml of medium containing 7.5 μg of polybrene in a 35 mm cell culture dish. Media was changed the following day and allowed to recover for 24h. The selection was performed using 2 μg/ml puromycin. Cells were then plated to 12-well plates for differentiation as described above. Puromycin was removed from the medium beginning on day 0 of differentiation.

### Generation of CRISPR/Cas9 KO in hAPCs

Isolation, proliferation, and differentiation of human adipocyte progenitor cells (hAPCs) (obtained from an anonymous male donor, approved ethical permit from the regional ethics board of Stockholm) were performed as previously described (51). *BCKDHA* single guide RNA (sgRNA) was designed and ordered from Integrated DNA Technologies (IDT) (sequence CGGUCCAUGACGCGGUAGAU). One day before the start of differentiation, hAPCs were transfected with sgRNA (final concentration of 1uM) that is complexed with Cas9 Nuclease (1,081,059, Integrated DNA Technologies) by electroporation using a Neon Transfection System (1700V, 20 ms, 1 pulse) and maintained in DMEM 10 mM HEPES (Gibco, 15,630-056), 10% fetal bovine serum (HyClone, SV30160.03), penicillin-streptomycin (50 μg/ml; Thermo Fisher Scientific, 15-140-122). Results were compared with TrueGuide sgRNA Negative Control (A35526, Thermofischer Scientific). Induction of differentiation started next for 13 days by adding an adipogenic cocktail consisting of insulin (5 mg/ml), 0.25 mM dexamethasone, 0.5 mM IBMX, transferrin (10 μg/ml; T8158, Sigma-Aldrich), 0.2 nM triiodothyronine (T6397, Sigma-Aldrich), and 10 mM rosiglitazone. Dexamethasone and IBMX were removed on Day 3, and rosiglitazone was removed on Day 10.

### Lipid droplet staining

For imaging differentiated human adipocytes were fixed in 4% PFA for 15 minutes at room temperature and washed three times with PBS. Lipid droplets and nuclei were stained with PBS containing BODIPY 493/503 (1:2500,#D3922, ThermoFisher) and Hoechst 33,342 (1:5000, #ab228551, Abcam) for 15 minutes. Cells were then washed four times with PBS. The images were acquired on a Nikon Ti2 multipoint confocal, equipped with a Kinetix sCMOS camera (Photometrics). The excitation wavelength used for imaging are: 405 and 477 nm and the emission filters are 438/24 and 515/30. Images were taken with z-stack with 2.5 μm step size. A 10X objective with a 1.5X eyepiece was used and it yields a total magnification of 15X.

### Insulin-stimulated glucose metabolism

Differentiated 3T3-L1 adipocytes were insulin-starved for 6h in 5 mM glucose DMEM + 0.5% FBS. Cells were then glucose-starved in Kreb’s Ringer Buffer (KRB) + 1.26 g/L sodium bicarbonate for 1h. Cells were labeled using 0.5 ml 5 mM [U-^13^C_6_]glucose KRB ± 100 nM insulin for 10 min before extraction for GC-MS analysis.

### Western blots

3T3-L1 adipocytes were lysed in ice-cold RIPA buffer with 1x protease inhibitor (Sigma-Aldrich). 25 μg total protein was separated on a 10% SDS-PAGE gel for BCKDHA and β-actin, while 50 μg total protein was loaded for ACAD8. The proteins were transferred to a nitrocellulose membrane and immunoblotted with anti-BCKDHA (Novus Biologicals NBP1-79616) (1:2500 dilution), anti-β-Actin (Cell Signaling 4970S) (1:10,000), anti-ACAD8 (Aviva Systems Biology OAAB06085) (1:1000 dilution). Specific signal was detected with horseradish peroxidase-conjugated secondary antibody goat anti-rabbit (1:2500-1:10,000) using SuperSignal West Pico Chemiluminescent Substrate (Thermo Scientific) and developed using Blue Devil Autoradiography film (Genesee Scientific) or Bio-Rad Chemidoc XRS + Imaging device.

For human adipocytes, the protein was separated on a 12% SDS-PAGE gel, transferred onto PVDF membranes, and immunoblotted with anti-BCKDHA (Novus Biologicals NBP1-79616) (1:10,000 dilution) and anti-calnexin (C5C9) (Cell Signaling, #2679).

### Extraction of metabolites for GC-MS analysis

For cell culture, polar metabolites and fatty acids were extracted using methanol/water/chloroform with [^2^H_31_]palmitate and norvaline as lipid and polar internal standards, respectively, and analyzed as previously described (52). Briefly, cells were washed twice with saline, quenched with −80 °C methanol and 4 °C water containing norvaline, scraped into Eppendorfs, and extracted with chloroform containing [^2^H_31_]Palmitate. After centrifugation, phases are dried separately. Samples are stored at −20 °C before analysis by GC-MS.

### GC-MS analysis

Dried polar metabolites were derivatized in 2% (w/v) methoxyamine hydrochloride (Thermo Scientific) in pyridine and incubated at 37 °C for 60 min. Samples were then silylated with N-tertbutyldimethylsilyl-N-methyltrifluoroacetamide (MTBSTFA) with 1% tert-butyldimethylchlorosilane (tBDMCS) (Regis Technologies) at 45 °C for 30 min. Polar derivatives were analyzed by GC-MS using a DB-35MS column (30m × 0.25 mm i.d. × 0.25 μm, Agilent J&W Scientific) installed in an Agilent 7890B gas chromatograph (GC) interfaced with an Agilent 5977A mass spectrometer (MS) with an XTR ion source. The dried lower chloroform phase was derivatized to form fatty acid methyl esters (FAMEs) *via* addition of 500 μl 2% H_2_SO_4_ in MeOH and incubation at 50 °C for 2 h. FAMEs were extracted *via* addition of 100 μl saturated salt solution and 2 500 μl hexane washes. These were analyzed using a Select FAME column (100m × 0.25 mm i.d.) installed in an Agilent 7890A GC interfaced with an Agilent 5975C MS using the following temperature program: 80 °C initial, increase by 20 °C/min to 170 °C, increase by 1 °C/min to 204 °C, then 20 °C/min to 250 °C and hold for 10 min. The percent isotopologue distribution of each fatty acid and polar metabolite was determined and corrected for natural abundance using in-house algorithms adapted from Fernandez *et al.* (53). Mole percent enrichment (MPE) was calculated *via* the following equation:

$$\frac{\sum_{i=1}^{n}M_{i}\cdot i}{n}$$

### Isotopomer spectral analysis (ISA) of fatty acids

Mass isotopomer distributions were determined by integrating metabolite ion fragments and correcting for natural abundance using in-house algorithms as previously described (14). The ISA compares a measured fatty acid isotopomer distribution to one that is simulated using a reaction network for palmitate synthesis, whereby 8 AcCoA molecules are consumed to form one palmitate molecule. Models were also generated for OCFA synthesis, whereby 1 PropCoA and 6 to 7 AcCoA molecules are consumed to form one OCFA as previously described (7). Parameters for the relative enrichment of the lipogenic AcCoA pool from a given [^13^C] tracer and the percentage of fatty acids that are *de novo* synthesized are extracted from a best-fit model using INCA v1.6 metabolic flux analysis software package (24). The 95% confidence intervals for both parameters were estimated by evaluating the sensitivity of the sum of squared residuals between measured and simulated fatty acid mass isotopomer distributions to small flux variations.

### Metabolic flux analysis

^13^C MFA was conducted using INCA, a software package based on the elementary metabolite unit (EMU) framework (24, 54). Fluxes through the metabolic network comprising of glycolysis, the pentose phosphate pathway, the TCA cycle, BCAA catabolism, and fatty acid synthesis and oxidation were estimated by minimizing the sum of squared residuals between experimental MIDs and extracellular fluxes using nonlinear least squares regression for differentiated 3T3-L1 adipocytes traced with [U-^13^C_6_]glucose, [U-^13^C_5_]valine, [U-^13^C_6_]leucine. The best global fit was found after estimating 20 times using random initial guesses for all fluxes in the metabolic network. A χ^2^ statistical test was applied to assess the goodness-of-fit using α of 0.01. The 95% confidence intervals for each flux in the metabolic network were estimated by sensitivity analysis due to minor flux variations (55). See Table S1 for metabolites used in the analysis, below for assumptions, and Tables S2 and S3 for raw flux values.

^13^ C metabolic flux analysis was conducted under the following assumptions.

• Cells were assumed to be at metabolic and isotopic steady state, *i.e.* intracellular free metabolite pool size and ^13^C enrichments are constant.
• Per well extracellular flux of glucose, lactate, glutamine, glutamate, alanine, valine, and leucine were assumed to be constant throughout the labeling experiments.
• Experimental replicates from all three tracers were entered into INCA v2.0 and used simultaneously to estimate fluxes for sgControl and sgBckdha, resulting in tighter confidence intervals.
• Cells were assumed to be terminally differentiated and did not divert resources towards biomass synthesis.
• Succinate and fumarate are structurally symmetric and can be enzymatically converted to their subsequent products in either configuration.
• Labeled CO_2_ release during decarboxylation reactions is diluted upon release and does not reincorporate during carboxylation reactions.
• Separate mitochondrial and cytosolic pools of aspartate, oxaloacetate, malate, fumarate, pyruvate, acetyl-CoA, glutamate, and glutamine were modeled with exchange fluxes for malate, aspartate, glutamate, and glutamine.
• Relative branching of glucose flux to the oxidative pentose phosphate pathway (PPP) relative to the glucose uptake flux was set to 0.3% (25).

### RNA isolation and quantitative RT-PCR analysis

3T3L1 adipocytes: Total RNA was purified from cultured cells using Trizol Reagent (Life Technologies) as per the manufacturer’s instructions. First-strand cDNA was synthesized from 0.5 μg of total RNA using a High-capacity cDNA Reverse Transcription Kit with RNase inhibitor (Applied Biosystems) according to the manufacturer’s instructions. Individual 10 μl SYBR Green real-time PCR reactions consisted of 2 μl of diluted cDNA, 5 μl of SYBR Green Supermix (Bio-Rad), and 1 μl of primer master mix containing each forward and reverse primer at 5 μM. For standardization of quantification, ribosomal RNA RPL27 was amplified simultaneously. The PCR was carried out on 96-well plates on a CFX Connect Real time System (Bio-Rad) using a three-stage program provided by the manufacturer: 95 °C hot-start for 3 min, 40 cycles of 95 °C for 10s and 60 °C for 30s, followed by a 0.5 °C/5 s meltcurve generation protocol. Gene-specific primers used are listed in Table S5.

Human adipocytes: Total RNA was purified using aNucleoSpin RNA isolation kit (#740955, Macherey-Nagel) and cDNA was synthesized using iScript cDNA synthesis (#1708891, BioRad). Expression of B2M and HPRT1 was used for normalization and gene-specific primers are listed in Table S6.

### RNA-seq library preparation

Cells were lysed in Trizol and total RNA was extracted per manufacturer’s instructions. Stranded RNA-Seq libraries were prepared from polyA-enriched mRNA using the Illumina Stranded mRNA library prep kit. Library construction and sequencing was performed by the University of California San Diego (UCSD) Institute for Genomic Medicine. Libraries were run as PE100 on a NovaSeq S4 to a depth of ∼25 million reads.

### RNA-seq analysis and gene set enrichment analysis

The Kallisto/Sleuth differential expression pipeline analysis was performed for paired triplicate samples of sgControl, sg*Bckdha*, and sg*Acad8*. The index was obtained from the Pachter lab Kallisto website “Ensembl Transcriptomes v96”, mus_musculus.tar.gz. Kallisto was run for paired-end read quantification with sequence-based bias correction (--bias) and 100 bootstraps (56). Normalized transcript abundances were further passed into sleuth using R, which were then aggregated to gene level (57). Ultimately, a pairwise Wald test was performed to compare sgControl v. sgBckdha and sgControl v. sgAcad8 using sleuth.

GSEA analysis using the KEGG pathway functional database was performed using the WEB-based GEne SeT AnaLysis Toolkit (WebGestalt). A rank score was calculated through the multiplication of the q-value with the sign of the fold change of sgBckdha and sgControl adipocytes. Only significantly differentially expressed genes (q < 0.05) were included in the analysis. Redundancy reduction of the enriched gene sets was set to “All.” The MSigDB Hallmarks pathway functional database was downloaded in gene symbol format (http://www.gsea-msigdb.org/gsea/msigdb/collections.jsp) (58, 59). As this is a human pathway database, the differentially expressed genes in the present study were first mapped to their human homologs using the publicly available mouse human genome homology database compiled by the mouse genome informatics organization (http://www.informatics.jax.org/downloads/reports/) (60). GSEA analysis was then conducted using the WebGestalt platform, using the hallmarks pathway set as a custom database, and the human homologs of the differentially expressed genes (q < 0.05) in this study and their associated rank score as the input gene list.

### Respirometry

Respirometry was conducted using a Seahorse XF96E Analyzer. For respiration studies, cells were plated at 5000 cells/well and maintained in 2 μg/ml puromycin-containing media until confluence was reached and differentiation was performed as described above. 8 days after differentiation was initiated, growth medium was replaced with DMEM (Sigma #5030) supplemented with 25 mM HEPES, 8 mM glucose, 2 mM glutamine, and 1 mM pyruvate. Mitochondrial respiration is calculated as the oxygen consumption rate sensitive to 1 μM rotenone and 2 μM antimycin A. Basal, maximal and ATP-linked respiration were calculated as previously described (61). Immediately after the experiment, cells were gently washed with PBS and either a) fixed with 4% paraformaldehyde and stained with CyQuant (Life Technologies) to determine cell number for normalization purposes or b) solubilized in RIPA buffer and quantified using BCA assay. Data from n = 3 independent experiments is depicted after normalization within each experiment.

### Statistical analyses

Statistical analysis of RNA seq data was carried out as detailed above. Metabolomic data in Figure 2 was analysed using MetaboAnalyst 5.0 where any missing data was input using k nearest neighbour, data was log-scaled and analysed using one-way ANOVA with *post hoc* analysis using Tukey’s HSD. Significance was considered with a FDR <0.05. Data was autoscaled for visualization in heatmaps. Statistical approach for other data is detailed in figure legends. All experiments were repeated at least 2 to 3 times and data from 1 representative experiment is shown unless otherwise specified. For analysis involving 2 groups, a two-tailed Student’s *t* test was performed. For analyses with 3 groups or more, a one-way ANOVA was performed as appropriate with *post hoc* analysis using Tukey’s HSD in GraphPad Prism. Statistical analysis was performed on all bar graphs displayed with the exception of 4A and S3A where error bars are confidence intervals and comparisons are deemed significant if bars are not overlapping. Statistical analysis was not performed on stacked bar plots (Fig. 4*D*) and XY line charts (Figs. 2*F* and 3*B*) but rather their corresponding bar graphs. Statistical significance was conveyed *via* a ∗ symbol for all significantly different comparisons between control and KO cells, and ^a,b,c^ to indicate a difference between sgBckdh guides. If a ∗ or ^a,b,c^ symbol was not shown, a *p* value of less than <0.05 was not found.

## Data availability

The RNA-seq data that support the findings of this study are available 10.13039/100000085GEO with accession number GSE205922, and any other data is available from the corresponding author upon reasonable request.

## Supporting information

This article contains supporting information.

## Conflict of interest

The authors declare that they have no conflicts of interest with the contents of this article.
