# Supplementary material for: Impaired branched-chain amino acid (BCAA) catabolism during adipocyte differentiation decreases glycolytic flux
Source: J Biol Chem. 2024 Nov 16;300(12):108004. doi: 10.1016/j.jbc.2024.108004 (PMC11667163; doi:10.1016/j.jbc.2024.108004)
Supplement: Supplementary Tables [file mmc1.pdf]

Supplementary Table 1: GC-MS metabolite fragment ions. Metabolites used in sgControl v. sgBckdha MFA model:

Metabolite fragment ions used for GC-MS analysis:

**Metabolite**	**Carbons**	**Formula**	**Mass (m/z)**
Pyruvate	123	$C_6H_{12}O_3NSi$	174
aKG	12345	$C_{14}H_{28}O_5NSi_2$	346
Fumarate	1234	$C_{12}H_{23}O_4Si_2$	287
Malate	1234	$C_{18}H_{39}O_5Si_3$	419
Glutamate	12345	$C_{19}H_{42}O_4NSi_3$	432
Glutamine	12345	$C_{19}H_{43}O_3N_2Si_3$	431
Citrate	123456	$C_{20}H_{39}O_6Si_3$	459
Palmitate	1-16	$C_{17}H_{34}O_2$	270
Pentadecanoic Acid	1-15	$C_{16}H_{32}O_2$	256
Heptadecanoic Acid	1-17	$C_{18}H_{36}O_2$	284
Leucine	23456	$C_{13}H_{32}O_1N_1Si_2$	274
Ketoisocaproate	123456	$C_9H_{18}O_3N_1Si_1$	216
Valine	12345	$C_{13}H_{30}O_2N_1Si_2$	288

Supplementary Table 2. MFA of 3T3-L1 sgControl. Full results of the ^{13}C-MFA model.

**3T3-L1 sgControl**	**Flux**	**95% Confidence Interval**	
**Pathway/Reaction**	**(nanomoles/well/hr)**	**Lower Bound**	**Upper Bound**
**Glycolysis**			
Glc.x -> G6P	188.03	184.41	191.68
G6P <-> F6P	187.43	183.81	191.08
G6P <-> F6P	94.24	0.00	Inf
F6P -> DHAP + GAP	187.83	184.21	191.48
DHAP <-> GAP	187.83	184.21	191.48
DHAP <-> GAP	0.00	0.00	Inf
GAP <-> 3PG	375.86	368.62	383.17
GAP <-> 3PG	86.70	0.00	Inf
3PG -> PEP	375.86	368.62	383.17
PEP -> Pyr.c	375.86	368.62	383.17
Pyr.c <-> Lac	264.19	254.49	273.68
Pyr.c <-> Lac	2.86	0.00	Inf
Lac -> Lac.x	264.19	254.49	273.68
Pyr.c -> Ala	13.97	8.98	15.58
Pyr.m -> Ala	0.00	0.00	4.88
Ala -> Ala.x	13.97	12.35	15.58
**Pentose Phosphate Pathway**			
G6P -> P5P + CO2	0.60	0.57	0.60
P5P + P5P <-> S7P + GAP	0.20	0.19	0.20
P5P + P5P <-> S7P + GAP	0.00	0.00	NaN
S7P + GAP <-> F6P + E4P	0.20	0.19	0.20
S7P + GAP <-> F6P + E4P	0.00	0.00	Inf
P5P + E4P <-> F6P + GAP	0.20	0.19	0.20
P5P + E4P <-> F6P + GAP	0.00	0.00	Inf
**Anaplerotic Reactions**			
Pyr.c -> Pyr.m	97.71	86.04	109.92
Pyr.m + CO2 -> Oac.m	58.99	51.32	67.54
Oac.c -> PEP + CO2	0.00	0.00	2.71
Mal.m -> Pyr.m + CO2	66.51	58.13	75.78
Mal.c -> Pyr.c + CO2	0.00	0.00	2.67
PropCoA + CO2 -> SucCoA	1.66	0.95	2.45
Gln.c <-> Glu.c	6.29	2.17	6.29
Gln.c <-> Glu.c	0.00	0.00	4.12
Glu.c -> Glu.x	0.27	0.20	0.34
Glu.c <-> Glu.m	-11.06	-16.15	-8.09

Glu.c <-> Glu.m	0.00	0.00	4.12
Glu.m <-> Akg.m	-11.22	-12.85	-8.32
Glu.m <-> Akg.m	0.00	0.00	5.15
Akg.c <-> Glu.c	-17.08	-19.00	-14.11
Akg.c <-> Glu.c	1240.00	0.00	Inf
Akg.m <-> Akg.c	0.00	-2.17	0.90
Akg.m <-> Akg.c	0.00	0.00	0.90
Akg.c + CO2 <-> Cit.c	17.08	14.12	18.90
Akg.c + CO2 <-> Cit.c	0.00	0.00	1.04
Glu.m <-> Gln.m	0.16	-3.97	0.62
Glu.m <-> Gln.m	14.44	12.05	17.57
Gln.x -> Gln.e	6.13	5.67	6.59
Gln.e -> Gln.c	38.92	38.42	39.42
Gln.c -> Gln.e	32.79	32.52	33.05
Gln.m <-> Gln.c	0.16	-3.97	0.62
Gln.m <-> Gln.c	54900.00	11.98	Inf
**TCA Cycle**			
Pyr.m -> AcCoA.m + CO2	105.22	92.85	118.16
AcCoAOther -> AcCoA.m	18.48	14.99	22.35
AcCoA.m + Oac.m -> Cit.m	140.10	122.65	158.49
Cit.m <-> Akg.m + CO2	31.20	27.33	35.10
Cit.m <-> Akg.m + CO2	0.00	0.00	0.79
Akg.m -> SucCoA + CO2	19.97	17.06	23.28
SucCoA -> Suc	21.63	18.25	25.45
Suc <-> Fum.m	21.63	18.25	25.45
Suc <-> Fum.m	0.00	0.00	Inf
Fum.m <-> Mal.m	21.63	18.25	25.45
Fum.m <-> Mal.m	553.87	0.00	Inf
Mal.m <-> Oac.m	653.51	-1260.00	1440.00
Mal.m <-> Oac.m	0.00	0.00	2010.00
Oac.m <-> Asp.m	572.40	-1440.00	1360.00
Oac.m <-> Asp.m	398.54	0.00	Inf
Mal.c <-> Oac.c	130.53	-1400.00	Inf
Mal.c <-> Oac.c	0.00	0.00	Inf
Oac.c <-> Asp.c	256.52	-1360.00	Inf
Oac.c <-> Asp.c	533.54	0.00	Inf
Asp.c -> Fum.c	828.92	0.00	Inf
Mal.c <-> Fum.c	-828.92	-Inf	0.00
Mal.c <-> Fum.c	1580.00	0.00	Inf
Mal.c <-> Mal.m	698.39	-1210.00	1390.00

Mal.c <-> Mal.m	86100.00	0.00	Inf
Asp.m <-> Asp.c	572.40	-1440.00	1360.00
Asp.m <-> Asp.c	0.00	0.00	Inf
**BCAA Catabolism**			
Leu.x -> Leu	9.37	9.05	9.69
Leu <-> KIC	5.47	4.71	6.29
Leu <-> KIC	0.34	0.00	Inf
KIC <-> IsoVCoA + CO2	5.47	4.71	6.29
KIC <-> IsoVCoA + CO2	0.17	0.07	0.65
IsoVCoA + CO2 <-> HMGCoA	5.47	4.71	6.29
IsoVCoA + CO2 <-> HMGCoA	0.17	0.00	1500000.00
HMGCoA -> AcCoA.m + AcCoA.m + AcCoA.m	5.47	4.71	6.29
LeuT -> Leu	3.93	3.74	4.13
Leu -> LeuP	7.84	6.90	8.73
Val.x -> Val	5.85	4.96	6.74
ValT -> Val	1.51	1.26	1.77
Val -> ValP	0.00	0.00	2.57
Val <-> IsoBCoA + CO2	7.36	5.16	8.48
Val <-> IsoBCoA + CO2	0.54	0.38	0.72
IsoBCoA <-> MMA	7.35	4.89	8.48
IsoBCoA <-> MMA	0.00	0.00	Inf
MMA -> PropCoA + CO2	7.35	4.89	8.48
**Fatty Acid Synthesis**			
Cit.m -> Cit.c	108.91	93.79	125.05
Cit.c -> AcCoA.c + Oac.c	125.99	110.17	142.64
AcCoA.c + AcCoA.c + AcCoA.c + AcCoA.c + AcCoA.c + AcCoA.c + AcCoA.c + AcCoA.c -> Palm.s	10.62	7.97	17.15
Palm.s -> Palm	10.62	7.97	17.15
Palm.d -> Palm	7.36	5.47	12.08
PropCoA + AcCoA.c + AcCoA.c + AcCoA.c + AcCoA.c + AcCoA.c + AcCoA.c -> C15FA.s	5.69	1.48	7.04
C15FA.s -> C15FA	5.69	1.48	7.04
C15FA.d -> C15FA	1.16	0.00	1.56
C15FA + AcCoA.c -> C17FA.s	6.85	1.01	8.49
C17FA.s -> C17FA	6.85	1.01	8.49
C17FA.d -> C17FA	2.22	0.00	2.82

IsoBCoA + AcCoA.c + AcCoA.c + AcCoA.c + AcCoA.c + AcCoA.c + AcCoA.c + AcCoA.c -> IsoC16.s	0.01	0.00	2.51
IsoC16.s -> IsoC16	0.01	0.00	2.51
IsoC16.d -> IsoC16	0.00	0.00	1.46
**Dilution/Mixing**			
0*Pyr.c -> Pyr.mnt	0.00	0.00	0.01
0*Pyr.m -> Pyr.mnt	1.00	0.99	1.00
0*Mal.c -> Mal.mnt	0.00	0.00	1.00
0*Mal.m -> Mal.mnt	1.00	0.00	1.00
0*Asp.c -> Asp.mnt	0.98	0.00	1.00
0*Asp.m -> Asp.mnt	0.02	0.00	1.00
0*Fum.m -> Fum.mnt	0.99	0.00	1.00
0*Fum.c -> Fum.mnt	0.01	0.00	1.00
0*Cit.m -> Cit.mnt	0.76	0.53	0.97
0*Cit.c -> Cit.mnt	0.24	0.03	0.47
0*Glu.m -> Glu.mnt	1.00	0.74	1.00
0*Glu.c -> Glu.mnt	0.00	0.00	0.26
0*Gln.m -> Gln.mnt	0.00	0.00	1.00
0*Gln.c -> Gln.mnt	1.00	0.00	1.00
0*Akg.m -> Akg.mnt	0.46	0.41	0.55
0*Akg.c -> Akg.mnt	0.54	0.45	0.59
Pyr.mnt -> Pyr.fix	1.00	1.00	1.00
Asp.mnt -> Asp.fix	1.00	1.00	1.00
Mal.mnt -> Mal.fix	1.00	1.00	1.00
Fum.mnt -> Fum.fix	1.00	1.00	1.00
Cit.mnt -> Cit.fix	1.00	1.00	1.00
Akg.mnt -> Akg.fix	1.00	1.00	1.00
Glu.mnt -> Glu.fix	1.00	1.00	1.00
Gln.mnt -> Gln.fix	1.00	1.00	1.00
SSR	774	637	785

Supplementary Table 3. MFA of 3T3-L1 sgBckdha. Full results of the ^{13}C-MFA model.

**3T3-L1 sgBCKDHa**	**Flux**	**95% Confidence Interval**	
**Pathway/Reaction**	**(nanomoles/well/hr)**	**Lower Bound**	**Upper Bound**
**Glycolysis**			
Glc.x -> G6P	146.44	142.50	150.42
G6P <-> F6P	145.96	142.02	149.94
G6P <-> F6P	338.25	0.00	Inf
F6P -> DHAP + GAP	146.28	142.34	150.26
DHAP <-> GAP	146.28	142.34	150.26
DHAP <-> GAP	0.00	0.00	Inf
GAP <-> 3PG	292.73	284.84	300.67
GAP <-> 3PG	655.91	0.00	Inf
3PG -> PEP	292.73	284.84	300.67
PEP -> Pyr.c	292.73	284.84	300.80
Pyr.c <-> Lac	139.59	129.18	149.83
Pyr.c <-> Lac	502.56	0.00	Inf
Lac -> Lac.x	139.59	129.18	149.83
Pyr.c -> Ala	6.54	0.76	7.64
Pyr.m -> Ala	0.00	0.00	5.73
Ala -> Ala.x	6.54	5.43	7.65
**Pentose Phosphate Pathway**			
G6P -> P5P + CO2	0.48	0.42	0.48
P5P + P5P <-> S7P + GAP	0.16	0.14	0.16
P5P + P5P <-> S7P + GAP	0.00	0.00	NaN
S7P + GAP <-> F6P + E4P	0.16	0.14	0.16
S7P + GAP <-> F6P + E4P	0.00	0.00	NaN
P5P + E4P <-> F6P + GAP	0.16	0.14	0.16
P5P + E4P <-> F6P + GAP	0.00	0.00	Inf
**Anaplerotic Reactions**			
Pyr.c -> Pyr.m	146.60	134.52	159.13
Pyr.m + CO2 -> Oac.m	84.28	73.05	95.51
Oac.c -> PEP + CO2	0.00	0.00	5.18
Mal.m -> Pyr.m + CO2	98.22	88.30	110.01
Mal.c -> Pyr.c + CO2	0.00	0.00	4.62
PropCoA + CO2 -> SucCoA	1.33	0.46	2.27
Gln.c <-> Glu.c	6.29	-2.72	6.29
Gln.c <-> Glu.c	0.00	0.00	9.01
Glu.c -> Glu.x	0.18	0.17	0.19
Glu.c <-> Glu.m	-18.00	-28.50	-11.68

Glu.c <-> Glu.m	0.00	0.00	9.01
Glu.m <-> Akg.m	-11.51	-21.98	-5.22
Glu.m <-> Akg.m	42.62	22.21	74.63
Akg.c <-> Glu.c	-24.11	-34.61	-17.79
Akg.c <-> Glu.c	11000.00	0.00	Inf
Akg.m <-> Akg.c	0.00	-9.01	2.36
Akg.m <-> Akg.c	0.00	0.00	9.01
Akg.c + CO2 <-> Cit.c	24.11	17.80	30.85
Akg.c + CO2 <-> Cit.c	0.00	0.00	1.47
Glu.m <-> Gln.m	-6.49	-15.52	-5.77
Glu.m <-> Gln.m	34.95	27.18	42.57
Gln.x -> Gln.e	12.78	12.06	13.50
Gln.e -> Gln.c	42.74	41.97	43.52
Gln.c -> Gln.e	29.96	29.58	30.35
Gln.m <-> Gln.c	-6.49	-15.52	-5.77
Gln.m <-> Gln.c	72000.00	27.18	Inf
**TCA Cycle**			
Pyr.m -> AcCoA.m + CO2	160.53	147.75	173.78
AcCoAOther -> AcCoA.m	19.29	15.58	23.24
AcCoA.m + Oac.m -> Cit.m	186.06	170.15	202.61
Cit.m <-> Akg.m + CO2	55.55	48.94	62.80
Cit.m <-> Akg.m + CO2	0.00	0.00	2.38
Akg.m -> SucCoA + CO2	44.04	39.60	48.97
SucCoA -> Suc	45.37	40.55	50.72
Suc <-> Fum.m	45.37	40.55	50.72
Suc <-> Fum.m	0.01	0.00	Inf
Fum.m <-> Mal.m	45.37	40.55	50.72
Fum.m <-> Mal.m	83.23	0.00	Inf
Mal.m <-> Oac.m	101.78	-30700.00	1080.00
Mal.m <-> Oac.m	1440.00	0.00	Inf
Oac.m <-> Asp.m	0.00	-31300.00	940.84
Oac.m <-> Asp.m	0.00	0.00	Inf
Mal.c <-> Oac.c	0.00	-31600.00	Inf
Mal.c <-> Oac.c	0.00	0.00	Inf
Oac.c <-> Asp.c	154.62	-31200.00	Inf
Oac.c <-> Asp.c	51.29	0.00	Inf
Asp.c -> Fum.c	154.62	0.00	Inf
Mal.c <-> Fum.c	-154.62	-Inf	0.00
Mal.c <-> Fum.c	4790.00	0.00	NaN
Mal.c <-> Mal.m	154.62	-31100.00	30500.00

Mal.c <-> Mal.m	811.72	0.00	Inf
Asp.m <-> Asp.c	0.00	-31300.00	940.84
Asp.m <-> Asp.c	0.00	0.00	Inf
**BCAA Catabolism**			
Leu.x -> Leu	5.63	5.15	6.11
Leu <-> KIC	2.08	1.54	2.65
Leu <-> KIC	2.33	0.00	Inf
KIC <-> IsoVCoA + CO2	2.08	1.54	2.65
KIC <-> IsoVCoA + CO2	0.05	0.00	0.23
IsoVCoA + CO2 <-> HMGCoA	2.08	1.54	2.65
IsoVCoA + CO2 <-> HMGCoA	1.34	0.00	920000.00
HMGCoA -> AcCoA.m + AcCoA.m + AcCoA.m	2.08	1.54	2.65
LeuT -> Leu	1.37	1.24	1.52
Leu -> LeuP	4.93	4.10	5.73
Val.x -> Val	3.33	2.86	3.79
ValT -> Val	0.69	0.58	0.81
Val -> ValP	0.00	0.00	3.17
Val <-> IsoBCoA + CO2	4.02	2.67	4.58
Val <-> IsoBCoA + CO2	0.30	0.21	0.39
IsoBCoA <-> MMA	4.01	1.00	4.58
IsoBCoA <-> MMA	0.00	0.00	Inf
MMA -> PropCoA + CO2	4.01	1.00	4.58
**Fatty Acid Synthesis**			
Cit.m -> Cit.c	130.51	115.67	145.95
Cit.c -> AcCoA.c + Oac.c	154.62	140.14	169.69
AcCoA.c + AcCoA.c + AcCoA.c + AcCoA.c + AcCoA.c + AcCoA.c + AcCoA.c + AcCoA.c -> Palm.s	16.85	14.52	20.99
Palm.s -> Palm	16.85	14.52	20.99
Palm.d -> Palm	7.31	6.24	8.97
PropCoA + AcCoA.c + AcCoA.c + AcCoA.c + AcCoA.c + AcCoA.c + AcCoA.c -> C15FA.s	2.68	0.00	3.73
C15FA.s -> C15FA	2.68	0.00	3.73
C15FA.d -> C15FA	1.04	0.00	1.64
C15FA + AcCoA.c -> C17FA.s	3.73	1.20	5.25
C17FA.s -> C17FA	3.73	1.20	5.25
C17FA.d -> C17FA	4.11	1.03	6.00

IsoBCoA + AcCoA.c + AcCoA.c + AcCoA.c + AcCoA.c + AcCoA.c + AcCoA.c + AcCoA.c -> IsoC16.s	0.00	0.00	3.17
IsoC16.s -> IsoC16	0.00	0.00	3.17
IsoC16.d -> IsoC16	0.00	0.00	Inf
**Dilution/Mixing**			
0*Pyr.c -> Pyr.mnt	0.00	0.00	0.02
0*Pyr.m -> Pyr.mnt	1.00	0.98	1.00
0*Mal.c -> Mal.mnt	0.00	0.00	1.00
0*Mal.m -> Mal.mnt	1.00	0.00	1.00
0*Asp.c -> Asp.mnt	0.90	0.00	1.00
0*Asp.m -> Asp.mnt	0.10	0.00	1.00
0*Fum.m -> Fum.mnt	0.03	0.00	1.00
0*Fum.c -> Fum.mnt	0.97	0.00	1.00
0*Cit.m -> Cit.mnt	0.57	0.33	0.75
0*Cit.c -> Cit.mnt	0.43	0.25	0.67
0*Glu.m -> Glu.mnt	1.00	0.67	1.00
0*Glu.c -> Glu.mnt	0.00	0.00	0.33
0*Gln.m -> Gln.mnt	0.00	0.00	1.00
0*Gln.c -> Gln.mnt	1.00	0.00	1.00
0*Akg.m -> Akg.mnt	0.63	0.48	0.81
0*Akg.c -> Akg.mnt	0.37	0.19	0.52
Pyr.mnt -> Pyr.fix	1.00	1.00	1.00
Asp.mnt -> Asp.fix	1.00	1.00	1.00
Mal.mnt -> Mal.fix	1.00	1.00	1.00
Fum.mnt -> Fum.fix	1.00	1.00	1.00
Cit.mnt -> Cit.fix	1.00	1.00	1.00
Akg.mnt -> Akg.fix	1.00	1.00	1.00
Glu.mnt -> Glu.fix	1.00	1.00	1.00
Gln.mnt -> Gln.fix	1.00	1.00	1.00
SSR	617	580	721

Supplementary Table 4. CRISPR/Cas9 target sequences. Sense and anti-sense single-guide sequences of Control, Bckdha, and Acad8 guides used in pooled cell culture experiments

Guide Target	Sense	Anti-sense
Control guide	CACCGGCCGTGTTGCTGGATACGCC	AAACGGCGTATCCAGCAACACGGCC
Bckdha guide 1	CACCGCAGCGAAATTGAAACCGGCG	AAACCGCCGGTTTCAATTTCGCTGC
Bckdha guide 2	CACCGTGAGGGATCTGCGTGGCCAG	AAACCTGGCCACGCAGATCCCTCAC
Bckdha guide 3	CACCGCATGACCAACTATGGCGAGG	AAACCCTCGCCATAGTTGGTCATGC
Acad8 guide 1	CACCGCCTTCCGCATCACATCCACA	AAACTGTGGATGTGATGCGGAAGGC
Acad8 guide 2	CACCGGCCAACAGGATTGGGACCG	AAACCGGTCCCAATCCTGTTGGCC
Acad8 guide 3	CACCGAGGTGAGTCAGACATCTATG	AAACCATAGATGTCTGACTCACCTC

Supplementary Table 5. Primer sequences. Forward and reverse sequences of primers used to quantify expression of genes using quantitative RT-qPCR.

Gene name	Forward Sequence	Reverse Sequence
18S rRNA	AGTCCCTGCCCTTTGTACACA	CGATCCGAGGGCCTCACTA
Adiponectin (*AdipoQ*)	GACACCAAAAGGGCTCAGGA	GCCCTTCAGCTCCTGTCATT
Carbohydrate response element binding protein (*Chrebp*)	CACTCAGGGAATACACGCCTAC	ATCTTGGTCTTAGGGTCTTCA
Fatty acid binding protein 4 (*Fabp4*)	AGAAGTGGGAGTGGGCTTTG	CCAGCTTGTCACCATCTCGT
Glucose transporter type 4 (*Glut4*)	AGCCTCTGATCATCGCAGTG	ACTAAGAGCACCGAGACCAAC
Peroxisome Proliferator-Activated Receptor Gamma Coactivator 1-Alpha (Pgc1a)	CCCTGCCATTGTTAAGACC	TGCTGCTGTTCCTGTTTTC
Peroxisome proliferator-activated receptor γ (*PPARg*)	TTCGCTGATGCACTGCCTAT	ACAGACTCGGCACTCAATGG

Supplementary Table 6. Primer sequences used for human adipocytes.

Gene name	Forward Sequence	Reverse Sequence
BCKDHA	GGTGTGCTGATGTATCGGGAC	CTTGCAGCCGTAGTGGACA
PLIN-1	TGTGCAATGCCTATGAGAAGG	AGGGCGGGGATCTTTTCCT
CEBPb	GTTCATGCAACGCCTGGTG	CAGTCCGCCTCGTAGTAGAA
CEBPa	AGCCTTGTTTGTACTGTATG	AAAATGGTGGTTTAGCAGAG
FABP4	ACTOGOCCAGGAATTIGACG	CTCGTGGAAGTGACGCCTT
ACLY	ATCGGTTCAAGTATGCTCGGG	GACCAAGTTTTCCACGACGTT
ACACA	ATGTTGGCTTGCACCTAGTA	CCCCAAAGCGAGTAACAAATTCT
FASN	TGGCTICATAGOTGACTTCCA	AAGGACCTGTCTAGGTTTGATGC
HPRT1	CCTGGCGTCGTGATTAGTGAT	AGACGTTCAGTCCTGTCCATAA
B2M	AAGGACTGGTCTTTCTATCTC	GATCCCACTTAACTATCTTGG

Supplementary Table 7. Correlation analysis of genes enriched in KEGG glycolysis geneset with *BCKDHA* as determined by gene vs genlist analysis in Correlation AnalyzeR.

Gene	Correlation Value	p value
PFKL	0.728	1.72E-07
PIGQ	0.685	2.47E-07
ALDOA	0.649	3.31E-07
HK2	0.412	2.22E-06
PGAM1	0.298	6.75E-06
GAPDH	0.276	8.67E-06
ENO1	0.263	1.01E-05
TPI1	0.234	1.47E-05
LDHA	0.216	1.89E-05
PGK1	0.166	4.29E-05
PKM	0.0605	0.0008971
PGM2	-0.148	6.10E-05
