# Supplementary material for: Impaired branched-chain amino acid (BCAA) catabolism during adipocyte differentiation decreases glycolytic flux
Source: J Biol Chem. 2024 Nov 16;300(12):108004. doi: 10.1016/j.jbc.2024.108004 (PMC11667163; doi:10.1016/j.jbc.2024.108004)

Supplementary Figure 1

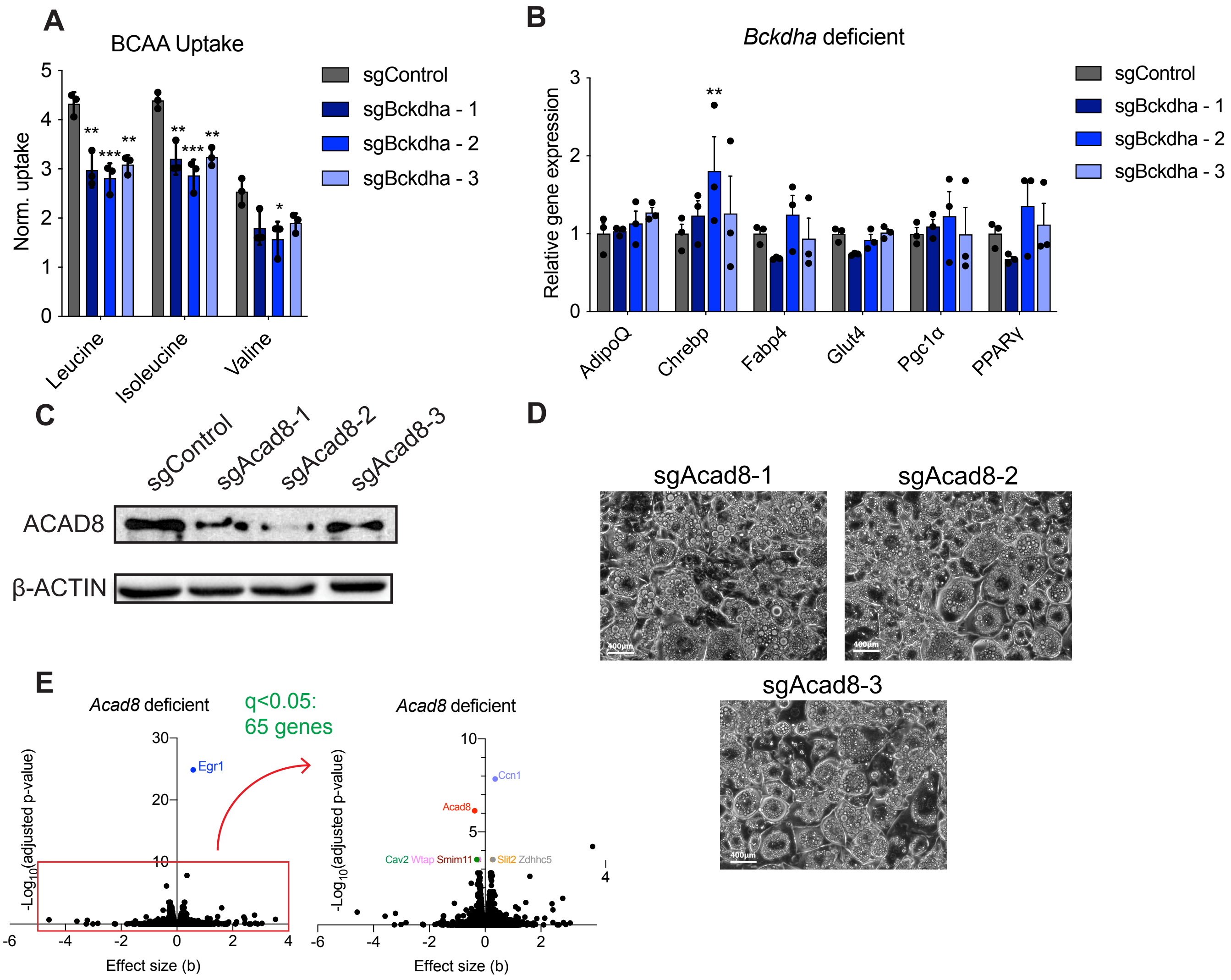

Supplementary Figure 2

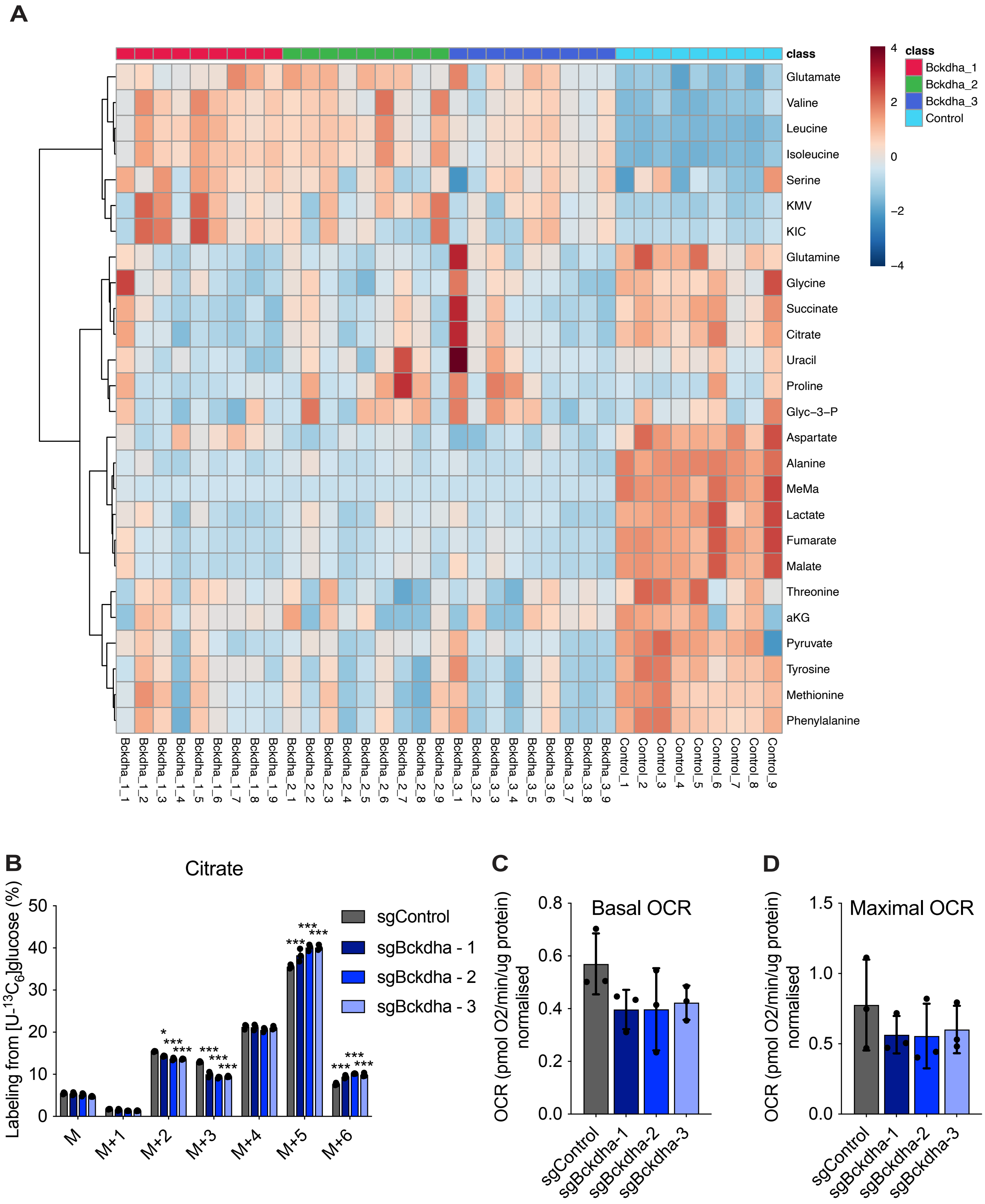

Supplementary Figure 3

A

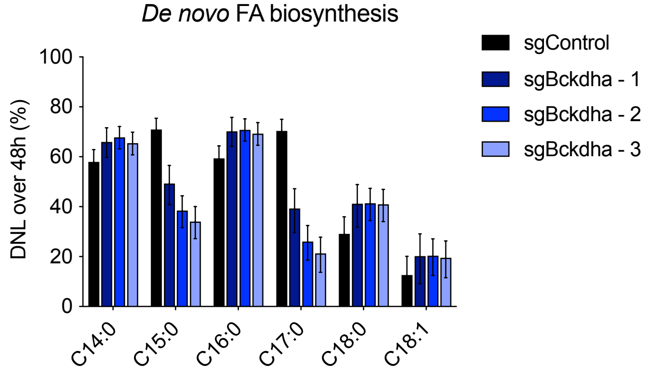

Supplementary Figure 4

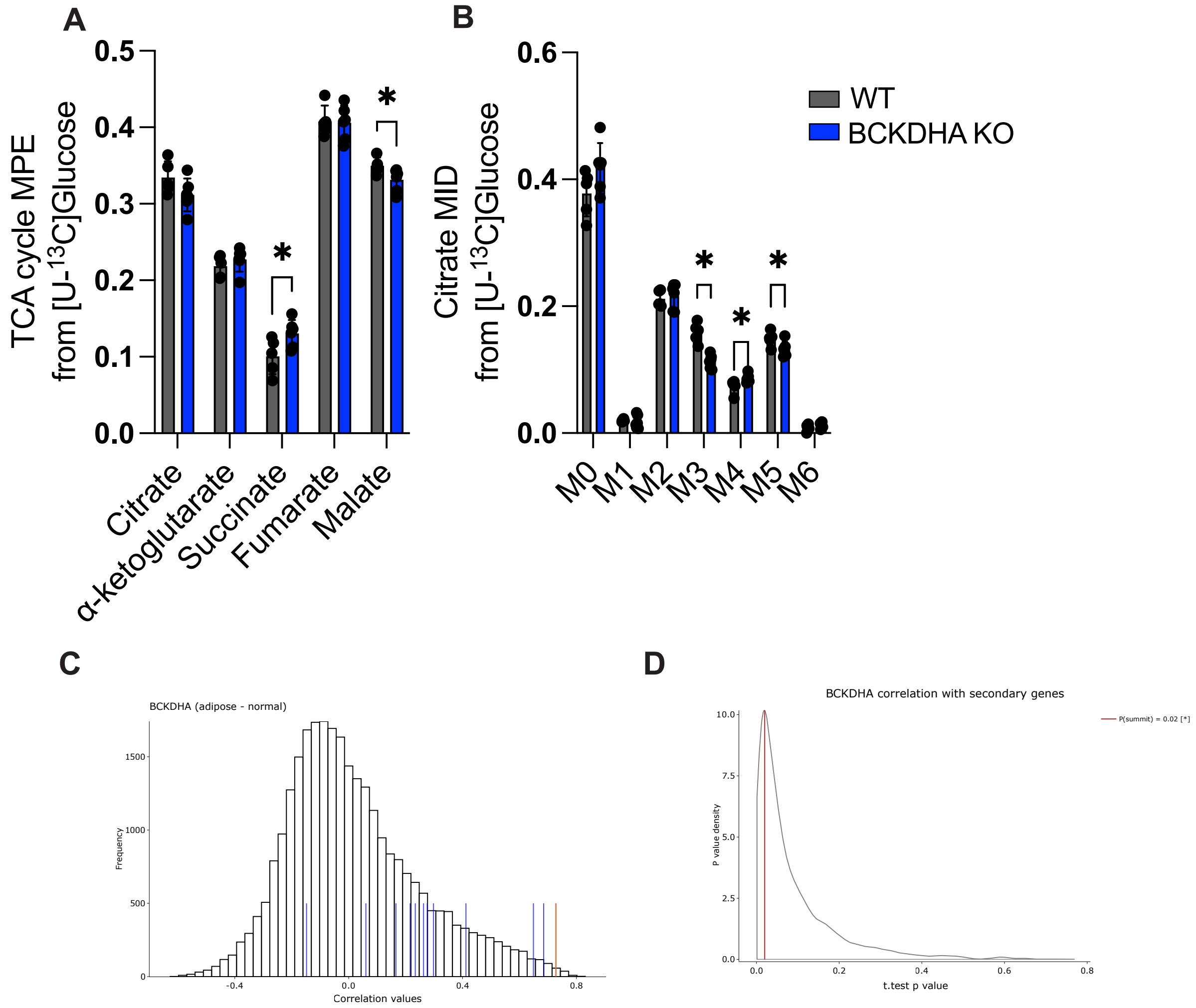

Supplement: Supplementary Figures [file mmc2.pdf]
