# Supplementary material for: Impaired branched-chain amino acid (BCAA) catabolism during adipocyte differentiation decreases glycolytic flux
Source: J Biol Chem. 2024 Nov 16;300(12):108004. doi: 10.1016/j.jbc.2024.108004 (PMC11667163; doi:10.1016/j.jbc.2024.108004)
Supplement: Supplementary Figure Legends [file mmc3.docx]

**Supplementary Figures**

**Supplementary figure 1.**

A. Normalized relative uptake of leucine, isoleucine, and valine in Control and *Bckdha*-deficient adipocytes. B. Expression of adipocyte differentiation markers normalized to sgControl. C. Western blot of ACAD8 and β-ACTIN. D. Brightfield images of differentiated Control and sgAcad8 adipocytes 7 days post-induction of differentiation (scale bar = 400μm). E. Volcano plots of differentially expressed genes in *Acad8*-deficient adipocytes compared to Control adipocytes. Data are presented as means ± SD (A-B) with three cellular replicates. Each parameter was analysed via one-way ANOVA with Tukeys post-hoc analysis comparing all group means. Significance in all is compared to sgControl. Results are depicted from one representative experiment which was repeated independently at least three times.

*p<0.05, **p<0.01, ***p<0.001.

**Supplementary Figure 2.**

A. Heat map of the abundance of intracellular metabolites in *Bckdha*-deficient adipocytes showing each replicate sample. B. Citrate mass isotopomer distribution (MID) after 48 hours in [U-^13^C_6_]glucose. Results are depicted from one representative experiment which was repeated independently at least three times. Each parameter was analysed via one-way ANOVA with Tukeys post-hoc analysis to compar each groups means. C-D. Basal and maximal oxygen consumption rates in Bckdha deficient adipocytes. n=3 experiments internally normalized to sgControl, analysed via one-way ANOVA with Tukeys post-hoc analysis. *p<0.05, **p<0.01, ***p<0.001.

**Supplementary figure 3.**

A. Percent of fatty acids derived from *de novo* lipogenesis in Control and *Bckdha*-deficient 3T3-L1 adipocytes over 48 hours obtained via isotopomer spectral analysis (ISA). Data is shown as means with 95% confidence interval (C.I.). Significance is denoted as non-overlapping 95% C.I.

**Supplementary figure 4.**

Characteristaion of [U^13^C] glucose utilisation in control and BCKDHA knock-out human adipocytes. A. Mole percent enrichment (MPE) of TCA cycle intermediates after 24 hours of incubation with [U-^13^C_6_]glucose. B. Citrate mass isotopomer distribution (MID) from [U-^13^C_6_]glucose. C. Histogram showing the location of the input genes within the correlation value distribution of *BCKDHA.* D. Density plot generated by permutation testing where the summit represents the empirically determined significance of *BCKDHA* with the input gene list.

For A-B. Data are presented as means ± SD with three cellular replicates. Results are depicted from one representative experiment which was repeated independently at least three times. Two-tailed students t-test was used to determine the p-value and shown as *p<0.05, **p<0.01, ***p<0.001.
